# Species-level, metagenomic and proteomic analysis of microbe-immune interactions in severe asthma

**DOI:** 10.1111/all.16269

**Published:** 2024-08-11

**Authors:** Maisha F Jabeen, Nicholas D Sanderson, Mariaenrica Tinè, Gillian Donachie, Clair Barber, Adnan Azim, Laurie CK Lau, Thomas Brown, Ian D Pavord, Anoop Chauhan, Paul Klenerman, Teresa L Street, Emanuele Marchi, Peter H Howarth, Timothy SC Hinks

**Affiliations:** 1 Respiratory Medicine Unit, Experimental Medicine Division, Nuffield Department of Medicine, University of Oxford, John Radcliffe Hospital, Oxford, UK; 2 Nuffield Department of Clinical Medicine, University of Oxford, John Radcliffe Hospital, Oxford, UK; 3 National Institute for Health Research Oxford Biomedical Research Centre, John Radcliffe Hospital, Oxford, UK; 4 Peter Medawar Building for Pathogen Research and Translational Gastroenterology Unit, Nuffield Department of Clinical Medicine, University of Oxford, Oxford, UK; 5 Department of Cardiac, Thoracic, Vascular Sciences and Public Health, University of Padova, 35128 Padova, Italy; 6 Clinical and Experimental Sciences, University of Southampton Faculty of Medicine, Sir Henry Wellcome Laboratories and NIHR Southampton Respiratory Biomedical Research Unit, Southampton University, Southampton, UK; 7 Portsmouth Hospitals NHS Trust, Portsmouth, UK

**Keywords:** Asthma, bacteria, cytokine, microbiome, sputum

## Abstract

**Background:**

The airway microbiome in severe asthma has not been characterised at species-level by metagenomic sequencing, nor have the relationships between specific species and mucosal immune responses in ‘type-2 low’, neutrophilic asthma been defined. We performed an integrated species-level metagenomic data with inflammatory mediators to characterise prevalence of dominant potentially pathogenic organisms and host immune responses.

**Methods:**

Sputum and nasal lavage samples were analysed using long-read metagenomic sequencing with Nanopore and qPCR in two cross-sectional adult severe asthma cohorts, Wessex (n=66) and Oxford (n=30). We integrated species-level data with clinical parameters and 39 selected airway proteins measured by immunoassay and O-link.

**Results:**

The sputum microbiome in health and mild asthma displayed comparable microbial diversity. By contrast, 23% (19/81) of severe asthma microbiomes were dominated by a single respiratory pathogen, namely *H. influenzae* (n=10), *M. catarrhalis* (n=4), *S. pneumoniae* (n=4) and *P. aeruginosa* (n=1). Neutrophilic asthma was associated with *H. influenzae, M. catarrhalis, S. pneumoniae* and *T. whipplei* with elevated type-1 cytokines and proteases; eosinophilic asthma with higher *M. catarrhalis*, but lower *H. influenzae*, and *S. pneumoniae* abundance. *H. influenzae* load correlated with Eosinophil Cationic Protein, elastase and IL-10. *R. mucilaginosa* associated positively with IL-6 and negatively with FGF. Bayesian network analysis also revealed close and distinct relationships of *H. influenzae* and *M. catarrhalis* with type-1 airway inflammation. The microbiomes and cytokine milieu were distinct between upper and lower airways.

**Conclusions:**

This species-level integrated analysis reveals central, but distinct associations between potentially pathogenic bacteria and airways inflammation in severe asthma.

## Introduction

Asthma is a complex, heterogenous disease. Recognising ‘treatable traits’ ascribed to specific pathophysiological subsets of severe asthma have supported significant therapeutic advances in ‘type-2 high’ severe asthma^1^; a phenotype characterised by sputum (≥3% on a cytospin)^2^ and blood eosinophilia, predictive of response to corticosteroid and anti-IL-5 therapy^3^. However, 30-50% of severe asthmatics have ‘type-2 low’ disease refractory to currently licensed biologic therapies or systemic corticosteroids^3^, constituting an important, poorly-understood, clinical phenotype. This encompasses neutrophilic (≥61% neutrophils on cytospin) and paucigranulocytic (non-neutrophilic or eosinophilic) asthma^3^.

In type-2 low disease, emerging data implicate bacterial airways infection with neutrophilic infiltration driven by innate and adaptive immune responses, and supporting long-term macrolide therapy^3,4^. Molecular microbiological approaches have described airway microbial profiles in severe asthma, but early studies were restricted by sequencing approaches unable to assign species level taxonomic identification. These reported reduced bacterial α-diversity, Proteobacterial enrichment, particularly *Haemophilus spp*. and *Moraxella spp*.^5–7^ and depletion of commensal organisms^8^. *H. influenzae* is the commonest pathogen detected in the lower airway^9–11^; it is linked to commensal depletion, neutrophilic inflammation and poor asthma outcomes^4^. However, prevalence of *H. influenzae* and other species is yet to be defined in severe asthma using a metagenomic approach resolved to species level.

Nanopore technology, in contrast to the Illumina platform, sequences DNA or RNA molecules as they pass through a protein nanopore in an electro-resistant membrane, without using PCR or chemical labelling. We recently demonstrated metagenomic sequencing using Nanopore offers rapid, species-level taxonomy from sputum samples^11^ at lower-cost, offering real-time data acquisition, and longer contiguous reads for *de novo* genome reconstruction. In our pilot study *H. influenzae* was commonly detected in severe neutrophilic asthma^11^. We thus hypothesised that a shift towards a pathogen-dominant airway microbiome is a common treatable trait in severe asthma, confined to the lower airway, with neutrophilic airways inflammation driven by type-1 cytokine responses. We aimed to define its prevalence, by inflammatory phenotype, in a large, adult, severe asthma population at species-level, using Nanopore metagenomic sequencing of induced sputum samples in stable disease, and discern inflammatory signals driving refractory airways inflammation through integrated proteomic analysis. By comparing the upper and lower airway microbiome and proteome in severe asthma we question the ‘one airway, one disease’ concept^12^.

## Methods

### Clinical cohorts

#### (1) Oxford Severe Asthma Cohort

Adult patients with American Thoracic Society/European Respiratory Society definition of severe asthma, on Global Initiative for Asthma (GINA) step 4-5 treatment (n=30) were recruited to the Oxford Airways Study between June 2018 and February 2020 (John Radcliffe Hospital, Oxford, UK, NHS Research Ethics approval 08/H0406/189). Participants underwent clinical phenotyping, sputum induction and phlebotomy during clinical stability. Hypertonic sputum induction and sputum differential cell counts were performed as previously described^13^ and sputum plugs stored at -80°C in sterile Brain Heart Infusion (BHI, Sigma-Aldrich, Dorset, UK) broth containing 10% glycerol. Sputum plugs were dispersed, and DNA extracted and sequenced (n=30) using established methods^11^.

#### (2) Wessex Severe Asthma Cohort^13^

Adult participants with severe asthma (n=51), mild/moderate asthma (n=10) and healthy controls (n=5) were recruited (University Hospital Southampton and Queen Alexandra Hospital Portsmouth). The study was approved by Southampton and South-West Hampshire Research Ethics Committee A (09/H0502/37) and study protocol described elsewhere^14^. Metagenomic sequencing was performed on induced sputum (n=66) and nasal lavage (NL) (n=17), and sequencing data integrated with existing cytokine data from sputum supernatant and nasal lavage^14^.

All participants provided written informed consent.

Sputum inflammatory subtypes were defined using validated criteria^2^. These were: eosinophilic ≥3% sputum eosinophils, neutrophilic ≥61% sputum neutrophils and <3% eosinophils, mixed granulocytic ≥61% sputum neutrophils and ≥3% eosinophils, paucigranulocytic <61% sputum neutrophils and <3% eosinophils.

### Molecular microbiology

Established methods^11^ were used for microbial DNA extraction from samples. Pathogen-specific qPCR and Nanopore sequencing performed and analysed as described in the online supplement.

### Measurement of inflammatory mediators

Existing data on 32 inflammatory mediators and 8 metalloproteinases were available from Hinks *et al*.^14^, measured by enzyme-linked immunosorbent assays and cytokine bead array (Luminex®) (see online supplement). In addition, O-link Uppsala, Sweden measured 92 inflammation-related proteins on sputum and nasal lavage using in-house Proseek Multiplex Inflammation I panel.

### Statistical and bioinformatic analyses

Statistical analysis were performed using Rstudio (Version 2022.12.0+353) and GraphPad Prism (Version 9). Data were logarithmically transformed if non-normal distribution. Parametric and nonparametric data are displayed as mean (SEM) and median (range), with comparisons between groups made using unpaired t-test or Mann-Whitney test, respectively. For all analyses, 2-tailed P-values <0.05 were considered significant. Where indicated, overall 5% significance level was adjusted for multiple comparisons with Benjamini-Hochberg procedure. Correlations were tested with Spearman’s *r* statistic, as described in the text. A full description of the bioinformatic analysis is provided in the online supplement and GitLab: https://gitlab.com/ModernisingMedicalMicrobiology/wessex_analysis.

Interconnectivity between clinical and immunological parameters alongside bacterial species was explored with Bayesian Network Analysis (BNA) (Genie 2.0; Decision Systems Laboratory, University of Pittsburgh, Pittsburgh, Pa), and is further described in online supplement.

## Results

Baseline participant characteristics are summarised in Table 1 (and Tables E1/E2, online supplement). The Wessex and Oxford cohorts were temporally and geographically separated, but comparable in their demographic features, baseline ICS use and lung function in severe asthma. The Wessex severe asthma cohort encompassed health, mild and severe asthma from a pre-biologic era, and hence a greater maintenance oral corticosteroid use in severe asthma participants, with a higher proportion of neutrophilic (25.5 vs. 20%) and paucigranulocytic (39 vs. 13.3%) sputum phenotypes. In addition, there was a greater proportion of current smokers (33 vs. 3.3%), but mean pack years smoked was <10. The Oxford cohort included participants with severe asthma alone, with a higher baseline blood eosinophil count (mean 0.4x10^9^/L vs. 0.2x10^9^/L) and eosinophilic sputum phenotype (36.7 vs. 25.5%). Overall, patients with severe asthma had high asthma control questionnaire (ACQ-5 in Oxford and ACQ-7 in Wessex cohort) scores indicative of uncontrolled disease (mean exceeding 1.5)^15^. Nanopore sequencing was performed on 96 sputum and 17 nasal lavage samples. A summary of sequencing statistics is provided in Supplementary Data File 1. In sputum samples median read length ranged from 1684 to 5490 bp (overall 2685 bp) and median bacterial reads per sample was 8099, and in nasal lavage samples median read length ranged from 2051 to 3904 bp (overall 2935 bp) and median bacterial reads per sample was 4010. A further integrated analysis, incorporating clinical and cytokine data, was performed in 66 participants from the Wessex cohort (Figure E1, online supplement).

### Nanopore sequencing defines distinct species-level metagenomes in severe asthma and identifies patients with a pathogen-dominant microbiome

**Figure 1:**
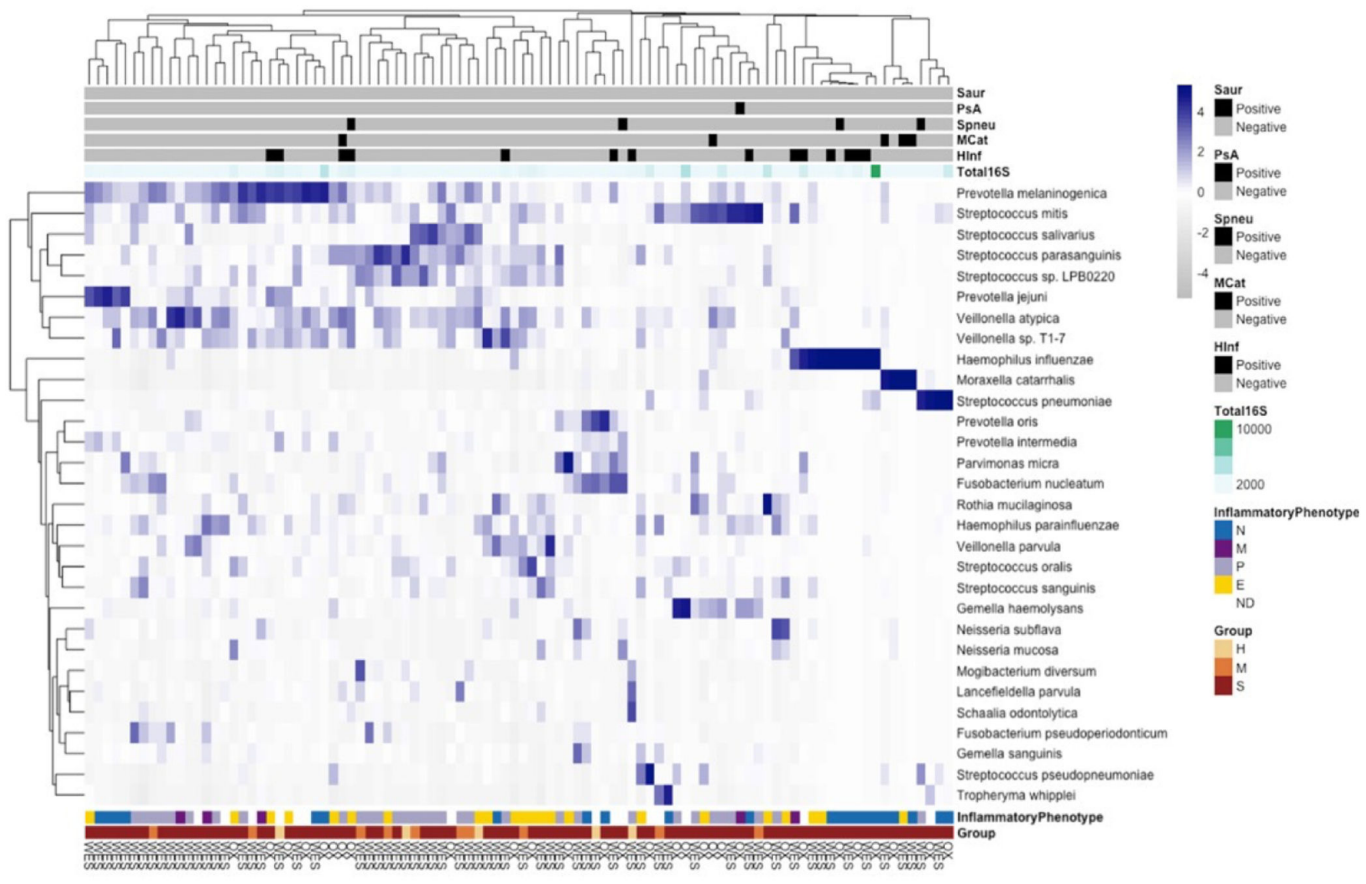
Relative abundance of species in sputum Heatmap of relative abundance of species from induced sputum sequenced samples using ONT; Z-scores, denoted by shade, represent the relative abundance of each taxon within each individual patient (columns). Cohorts include Wessex (WES; Healthy [H]: n=5, Mild [M]: n=10 and Severe asthma [S]: n=51) and Oxford (OX; Severe asthma[S]: n=30). Clustering by species (rows) and individuals (columns) using Euclidian distance performed independently on samples and most differentially abundant bacterial species. Sputum inflammatory phenotypes are eosinophilic (E), neutrophilic (N), mixed granulocytic (M) and paucigranulocytic (P). Positive pathogen specific PCR results (>1x106copies/ml) are indicated in black: *S. aureus* (Saur), *P. aeruginosa* (PsA), *S. pneumoniae* (Spneu), *M. catarrhalis* (Mcat) and *H. influenzae* (Hinf). Total eubacteria (16S) PCR load is denoted by shade (x106 copies/ml).

Z-scores of bacterial species with greatest variability in abundance across health (HC), mild (MA) and severe asthma (SA) are displayed in Figure 1 combining the Oxford and Wessex cohorts, and shown separately by cohort in Figure E2 (online supplement).

Hierarchical clustering revealed a cluster of 19/96 participants, each with severe asthma and skewed airway microbiome dominated by a single respiratory pathogen, namely *H. influenzae, M. catarrhalis* or *S. pneumoniae*. Remaining participants displayed greater microbial diversity with variable abundance of commensal organisms, predominantly phyla Actinobacteria, Bacteroidetes and Firmicutes. A principal component analysis (PCA) plot on normalised read counts filtered by abundance and variability (online supplement) for patients with severe asthma from the Wessex and Oxford cohorts is shown in Figure E3(b) (online supplement). There was no significant difference in the microbial composition between these groups (PERMANOVA = 0.19).

Differential abundance of species was tested across health (n=5), mild (n=10) and severe asthma (n=51) in the Wessex cohort only, which alone included these distinct groups. The most conserved species in HC sputum (HC vs MA/SA, log fold change [LFC] >1, adjusted P<0.05) include *Streptococcus viridans, Streptococcus sp A12, Streptococcus australis* and *Schaalia odontolytica* (Table E3, online supplement). In MA (MA vs HC/SA) 29 species, predominantly Firmicutes, were similarly identified, of which *Filifactor alocis, Mogibacterium diversum* and *Streptococcus sp. A12* were significantly enriched (Table E4, online supplement). SA (SA vs HC/MA) is associated with a marked compositional shift and greater abundance of species including *Rothia mucilaginosa, Rothia dentocariosa, H. influenzae* and *M. catarrhalis* (Table E5, online supplement), and relative depletion of oropharyngeal commensals (Table E5, online supplement).

We defined pathogen dominance – metagenomic dominance by a single airway pathogen – in an individual if a known respiratory pathogen (*H. influenzae, M. catarrhalis, S. pneumoniae* or *P. aeruginosa*, in this dataset) was the most abundant (%reads/total bacterial reads) species and the proportion of detected reads (Table E6) was ≥2-fold of the second ranked abundant species in the sample. Across two cohorts, pathogen-dominant microbiome was present during clinical stability in 23% of severe asthmatics (n=19/81). *H. influenzae* (n=10) was most prevalent, followed by *M. catarrhalis* (n=4) and *S. pneumoniae* (n=4). In most (n=18/19) samples, sequences covered ≥1/5^th^ of the reference genome, providing high confidence species-level classification. *P. aeruginosa* was detected in one individual with high proportion of total reads (73%) but lower genome coverage (5.1%). The relative abundance of these respiratory pathogens in individuals with a pathogen-dominant microbiome is shown in Sankey visualisations (Figure E4) and was significantly greater than other detected species (Wilcoxon rank-sum test, Benjamini-Hochberg [BH] correction FDR 0.05; Figure E4(B) Wessex, *H. influenzae* p = 6.3x10^-5^; Figure E4 (C) Wessex, *M. catarrhalis* p = 8.7x10^-4^; Figure E4 (C) Wessex, *S. pneumoniae* p = 0.01; Figure E4(F) Oxford, *H. influenzae* p = 3.8x10^-3^; Figure E5(a) Oxford, *S. pneumoniae* p = 0.01). Semiquantitative pathogen-specific qPCR results are shown in Figure 1/Table E6. A clinically significant positive result was identified as ≥1x10^6^ copies/ml, previously demonstrated to be longitudinally reproducible in COPD for identifying bacteria-associated neutrophilic inflammation^16^ and correlating with positive sputum culture in asthma^11^. By this definition a positive qPCR result was identified in all Oxford samples shown in Table E6 and 8/11 Wessex samples. Bacterial load was quantified with 16S qPCR (Figure 1) and was higher in Oxford (median 364x10^6^, IQR 823x10^6^ copies/ml) versus Wessex sputum samples (median 23x10^6^, IQR 83x10^6^ copies/ml).

### Sputum inflammatory phenotype is predictive of the presence of airway pathogens

**Figure 2:**
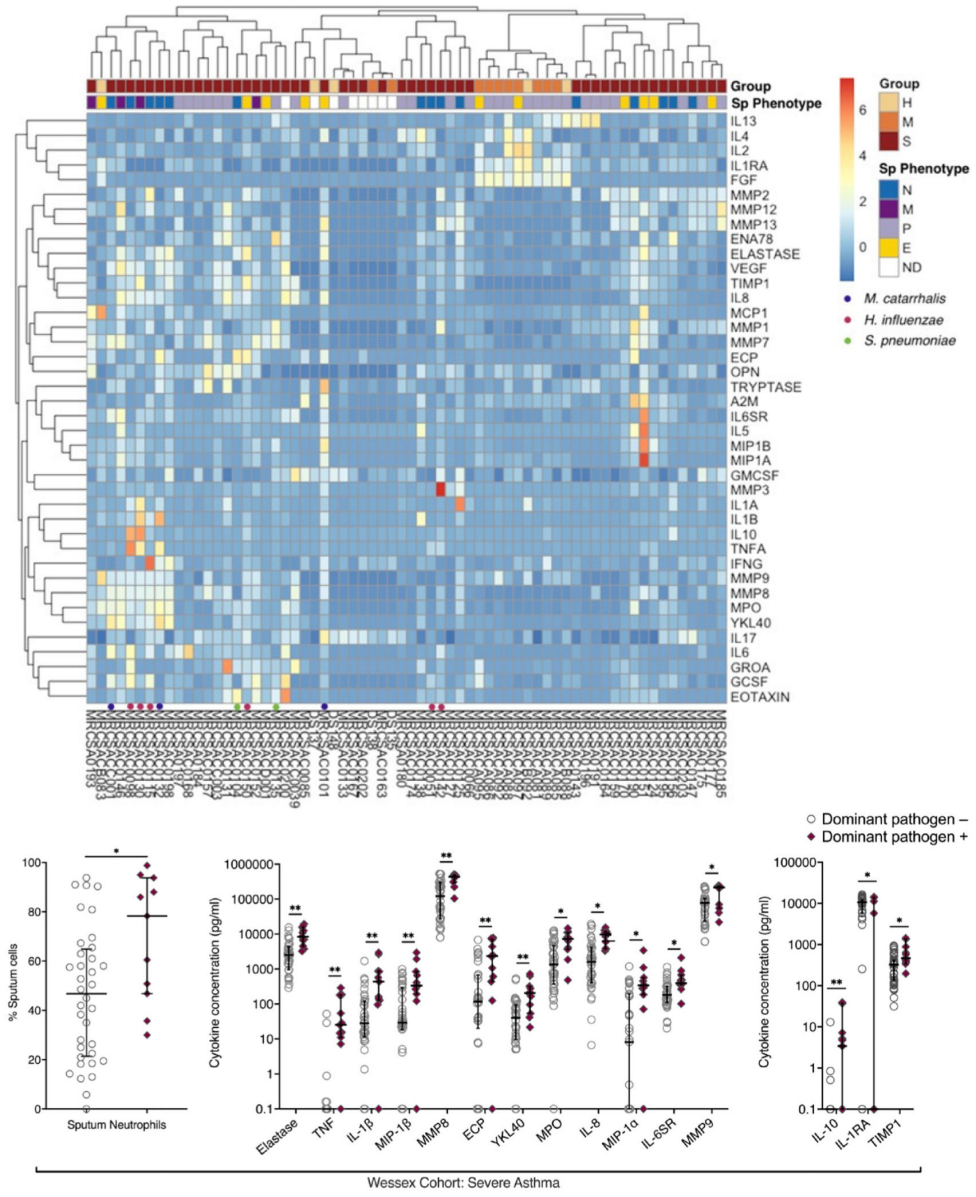
Integrated analyses of microbiome and inflammatory mediators in sputum Integrated cytokine analysis (A) Heatmap of scaled and centralised sputum cytokines in health, mild and severe asthma. Z-score centralised cytokine measurements denoted by shade. Independent clustering performed by subject (columns) and cytokine (rows). Clustering distances shown are representative of correlation distances. In severe asthma, presence of a dominant pathogenic organism on metagenomic sequencing is indicated as HI (*H. inf*), MC (*M. cat*) and SP (*S. pneu*); the pathogen-dominant microbiome in severe asthma (data only shown for participants with severe asthma) is associated with (B) sputum neutrophilia, (C) elevated type-1 cytokines and (D) anti-inflammatory mediators in sputum supernatant. Median/IQR shown −/+ Pathogen dominance ([C] Mann-Whitney test, [D]/[E] unpaired t-test, adjusted for multiple comparisons with BH procedure [FDR 0.05]. *P≤0.05, **P≤0.01).

A pathogen-dominant microbiome in severe asthma (Figure E4 and Figure E5, online supplement) was associated with a higher Proteobacteria:Firmicutes ratio (P<0.001, Figure E6, online supplement), consistent with previous reports^10,17^, but no significant differences were observed between SA (n=51) and HC (n=5) or MA (n=10), or across inflammatory phenotypes. This was consistent with Proteobacterial species *H. influenzae* and *M. catarrhalis* being the most prevalent airway pathogens in severe disease. A pattern of pathogen dominance of the microbiome was not observed in health or mild asthma. Airways neutrophilia was associated with *H. influenzae, M. catarrhalis, S. pneumoniae* and *P. aeruginosa* dominance in severe asthma sputum, with higher overall sputum neutrophil count (Figure 2B, P<0.05) and higher prevalence of neutrophilic asthma (Table E2 online supplement, P<0.05).

Defining bacterial species associated with severe asthma inflammatory phenotypes were sought (LFC>1, adjusted P<0.05). Consistent with the above findings, *H. influenzae, M. catarrhalis, T. whipplei* and *S. pneumoniae* were strongly associated with neutrophilic asthma, whilst commensal *Streptococcus spp*. (Table E7, online supplementary) including *S. salivarius, S. intermedius, S. oralis* were relatively depleted (LFC< -1, adjusted P<0.05). In paucigranulocytic disease, there was lower *M. catarrhalis, H. influenzae* and *T. whipplei* abundance (Table E7, online supplement). Eosinophilic asthma was characterised by high relative abundance of *M. catarrhalis, S. intermedius* and *V. parvula* and less *H. influenzae, S. pneumoniae* and *Actinomyces pacaensis*, a relatively newly-isolated Gram-positive human respiratory microbiome^18^ organism (Table E7, online supplementary). No organisms uniquely defined the mixed inflammatory phenotype.

### Integrated cytokine and metagenomic analyses

We integrated sputum metagenomes in the Wessex cohort (n=66) with existing cytokine data measured by immunoassay (n=40)^14^(Figure 2A). Unsupervised clustering identified HC and MA with higher expression of FGF, IL-1RA, IL-2 and IL-4 relative to SA clusters. Amongst SA two clusters were seen. One was associated with predominantly proinflammatory and type 1 cytokines (left) including IL-1α, IL-1β, TNF, IFN-γ, MMP8, MMP9, MPO, YKL40, IL-17, IL-6, elastase, ECP, IL-8, GROα, G-CSF, eotaxin and TIMP-1, typically in presence of a pathogen-dominant microbiome. The second cluster (right) represents a mixture of sputum inflammatory phenotypes, without infection, with high expression of MMPs.

**Figure 3:**
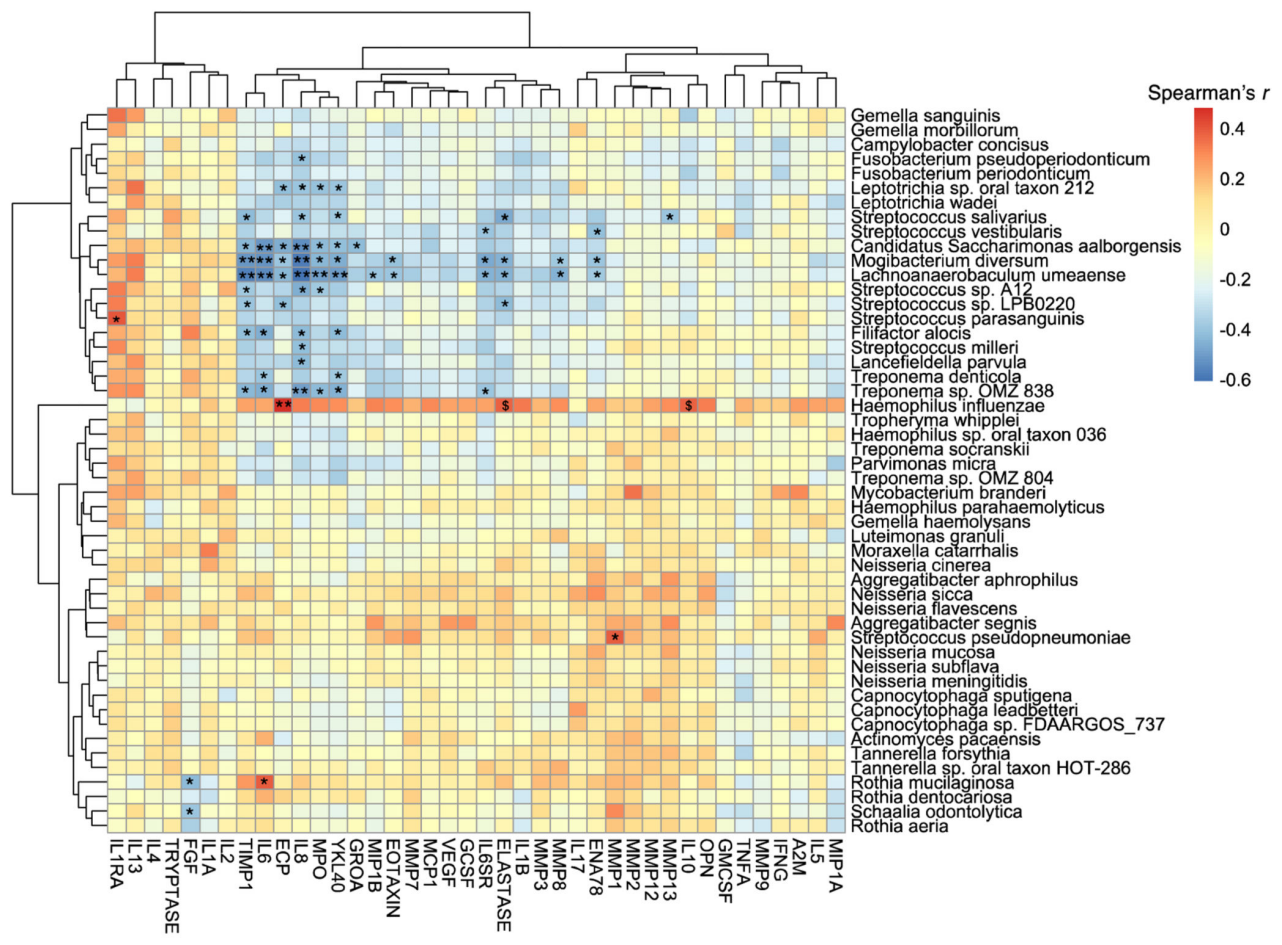
Heatmap displaying correlations between integrated sputum microbiome and cytokine data The 50 most variable bacterial species across all subjects were selected. Spearman correlation coefficients between species and cytokines denoted by shade. Independent clustering performed by cytokine (columns) and species (rows). Statistically significant correlations have been annotated as ^$^P=0.05, *P< 0.05 and **P<0.01. Adjustment for multiple comparisons was made using BH- procedure (FDR 0.05).

To further dissect the mucosal immune response to individual species we integrated these data, clustering (hierarchical) by species and by cytokine (Figure 3 and Supplementary Data), selecting the 50 species with greatest variance in abundance across subjects. The strongest positive correlations were between *H. influenzae* and Eosinophil Cationic Protein (ECP) (*r=*0.5, P≤0.007), IL-10 (*r*=0.4, P=0.05) and elastase (*r*=0.4, P=0.05); between *Rothia mucilaginosa* and IL-6 (*r*=0.4, P<0.05); between *Streptococcus pseudopneumoniae* and MMP1 (*r*=0.4, P<0.05); and between *Streptococcus parasanguinis* and IL-1RA (*r*=0.4, P<0.05) implying species-specific immune modulation. Negative correlations between predominantly Gram-positive oropharyngeal/respiratory commensals (top middle species cluster Figure 3) and pro-inflammatory cytokines were identified, exemplified using markers of neutrophilic inflammation –YKL40, MPO, IL-6 and IL-8 which correlated negatively (*r*<-0.4, P<0.05) with anaerobic Gram-positives *Mogibacterium diversum, Lachnoanaerobaculum umeaense* and the uncultured bacteria *Candidatus Saccharimonas aalborgensis*. TIMP-1 and MMP8 correlated negatively Gram-positive and negative commensals. Conversely *R. mucilaginosa*, a species with greatest overall detected reads in severe asthma (Table E5, online supplement), was linked to low FGF levels (*r*=-0.4, P< 0.05).

### Neutrophilic inflammation with pathogen dominance is accompanied by elevated type-1 cytokine and protease activity, elevated IL-10 and deficiency of IL-1RA in severe asthma

Using the above pragmatic sequencing definition of pathogen-dominance, we dichotomised severe asthma by presence or absence of this trait. A pathogen-dominant microbiome was associated with sputum neutrophilia (Figure 2B, P<0.05), significantly elevated type-1 cytokines and airway proteases, namely: elastase, TNF, IL-1βα, MIP-1β, MMP8, ECP, YKL40, MPO, MIP-1α, IL-6SR and MMP9 (Figure 2C, P<0.05 and Table E8, online supplementary). Of the immunomodulatory mediators IL-1RA was deficient, whereas TIMP-1 and IL-10 levels were higher in the presence of pathogen dominance (Figure 2D, P<0.05 and Table E8, online supplement).

### BNA in severe asthma

**Figure 4:**
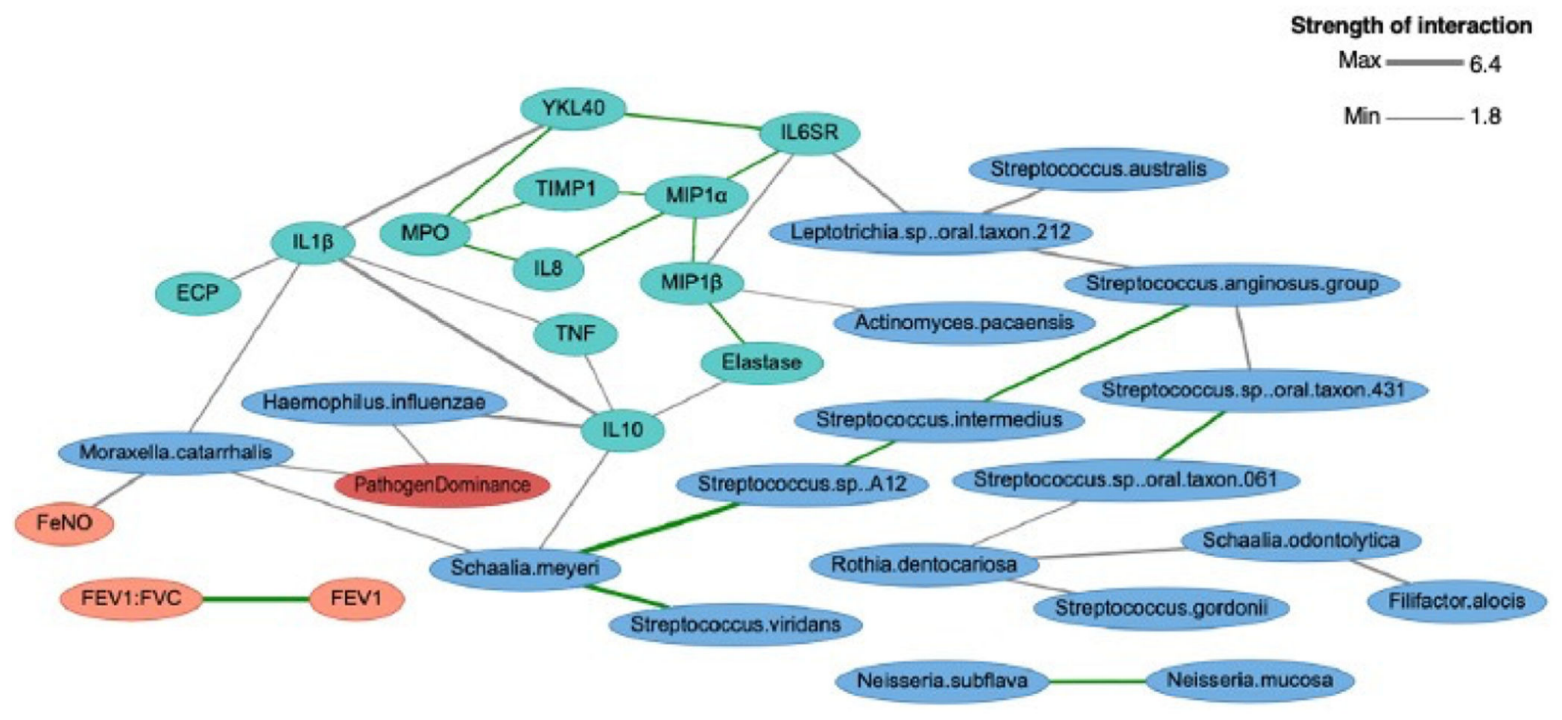
Integrated Bayesian Network Analysis Bayesian network showing strongest interactions between most differentially abundant bacterial species, inflammatory mediators, and clinical features in severe asthma (WES, n=51). Nodes are coloured by class: bacterial species (blue), infection status (red), inflammatory mediators (green) and clinical features (orange). Nodes without strong interactions have been excluded. Line thickness represents strength of interaction (Euclidean distance) as shown in the scale. Interactions with a positive linear correlation are shown with a green edge.

A Bayesian network is an acyclic directed graph in which variables are represented in nodes, and arcs represent the direct probabilistic dependencies between these^19^. In essence it an unbiased probabilistic method to explore how different parameters relate to each other, as the network structure models the interactions among the variable in the model. BNA was applied to cytokines significantly elevated in a pathogen-dominant microbiome (n=15), clinical parameters (n=20), and bacterial species with significant differential abundance (either increased or decreased) in severe asthma (n=25), in 51 severe asthmatics (Figure 4). The ‘clustering algorithm’ was applied^20,21^. The analysis retained 34/60 parameters in the model (Table E9). To further explore pair-wise interactions retained in this network, in a sperate secondary analysis, Pearson (between bacterial species) or Spearman (between cytokine) correlations were performed. Positive linear correlations are highlighted with a green edge. No negative correlations were found. This analysis provided an exploration of the inter-relationships between these key microbial and immune variables specifically in severe asthma, thereby supplementing the earlier integrated analyses performed in the full Wessex cohort.

Connectivity was observed between *H. influenzae*/*M. catarrhalis* abundance nodes and ‘pathogen dominance’, and between cytokines linked to pathogen dominance in earlier analyses. There was a non-linear relationship between *M. catarrhalis* and FeNO or IL-1β (Figure E7), and a mutually exclusive relationship between elevated FeNO and abundant *H. influenzae* (shown in Figure E8). In addition, connectivity appeared with positive correlations between related bacterial species, most notably species belonging to phylum Firmicutes, and *Neisseria* spp (Figure E9).

This approach highlighted associations between sputum IL-10 and *H. influenzae* abundance (Figure E7a, *r*=0.32, P<0.05), and IL-10 and TNF (Figure E7b, *r*=0.62, P<0.0001). The macrophage chemoattractant MIP1α node was highly connected within the network (Figure 4 and Figure E7, online supplement) specifically to complementary chemokine MIP1β (*r*=0.88, P<0.0001), alongside IL-6SR (*r*=0.65, P<0.0001), TIMP-1 (*r*=0.65, P<0.0001) and IL-8 (*r*=0.73, P<0.0001). Sputum YKL40 was strongly connected to MPO (*r*=0.92, P<0.0001), IL-6SR (*r*=0.75, P<0.0001) and IL-1β (*r*=0.63, P<0.0001). Neutrophilic mediators MPO and IL-8 strongly correlated (*r*=0.85, P<0.0001).

### Distinct microbiomes and cytokine milieus between upper and lower airways in severe asthma

**Figure 5:**
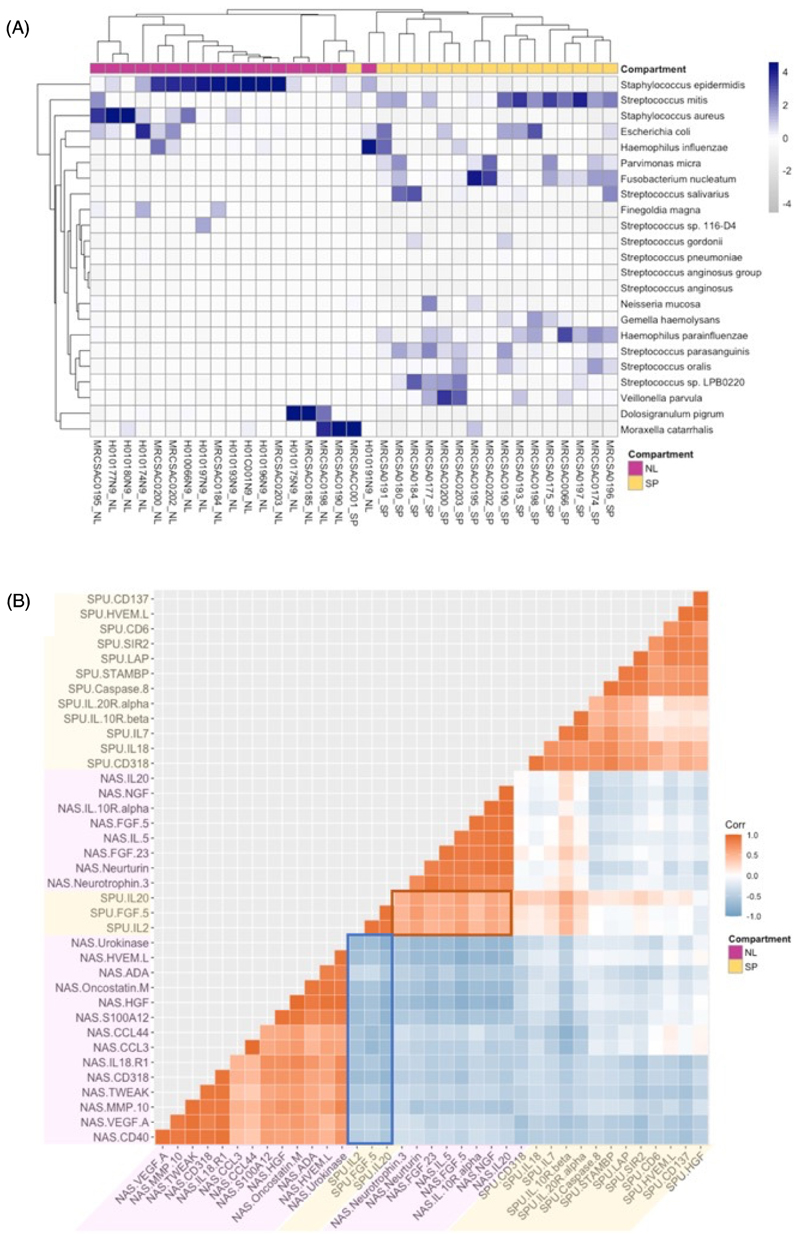
Upper and lower airway microbiome and cytokines (A) Heatmap of relative abundances of bacterial species on metagenomic sequencing of paired nasal lavage (NL) and sputum (SP) samples from severe asthmatics (n=17). Z-score denoted by shade. Independent clustering by samples (columns) and 23 most variable species (rows) using Euclidian distance. (B) Correlation matrix of the most significantly correlated (P<<0.001) proteins in paired nasal lavage (NAS) and sputum (SPU) cytokines (O-link). Significantly correlated pairs displayed (p<<0.001, adjusted for multiple comparisons with Benjamini–Hochberg procedure [FDR 0.05]).

Paired nasal lavage and sputum samples were collected in severe asthma (n=17). We consider nasal lavage as typical of part of the upper airway compartment, whereas, despite an inevitable component of oral contamination, sputum samples are more representative of the lower airways. Relative abundances of shared bacterial species between the upper and lower airways are shown in Figure 5A and Figure E10. Samples and species clustered distinctly by anatomical location (PCA plot shown in Supplementary Figure E11, PERMANOVA = 0.001). In severe asthma, nasal lavage was enriched in *S. epidermidis* and *S. aureus*, with higher abundance of *D. pigrum, M. catarrhalis* and *E. coli* compared to sputum. Sputum was enriched for Firmicutes (predominantly *Streptococcal spp*.) with greater relative abundance of *H. influenzae* and *H. parainfluenzae*.

The cytokine milieu in the upper and lower airway was compared using paired O-link proteomics from nasal lavage and sputum samples (Figure 5B), proteins with statistically significant correlations are shown. Proteins were discordant between nasal lavage and sputum compartments (that is, proteins most strongly correlate with others from the same compartment), with the exception of significant associations between sputum cytokines IL-20, FGF5 and IL-2 and specific nasal lavage proteins (described further in Supplementary Results).

## Discussion

Using long-read Nanopore sequencing we provide the first large-scale, species-level definition of the airway microbiome in severe asthma. We identified presence of a pathogen-dominant microbiome, with dominance of a single pathogenic bacterial species, and associated mucosal inflammation, in 20-30% of individuals during stable disease. Such dominance with a limited repertoire of species: *H. influenzae* being the most prevalent, followed by *M. catarrhalis* and *S. pneumoniae*. Whilst metagenomic measures do not imply bacterial tissue invasion, we identified strong correlation between dominance of potentially pathogenic bacteria and evidence of airway neutrophilia and mucosal inflammation beyond that expected by simple commensal colonisation. Using novel supervised and unsupervised approaches our integration of species-level metagenomics with cytokine and protein datasets shows consistent evidence of type-1 inflammatory responses accompanied by neutrophilia in presence of a pathogen-dominant microbiome. This coincides with commensal depletion (largely Firmicutes), previously linked to worse asthma outcomes^4^. Commensal organisms detected in induced sputum showed an expected overlap with oropharyngeal flora^22^. Our results are consistent with and build upon findings of previous studies^4,8,10^. Finally, we demonstrate that lower (sputum) airway microbiome remains distinct from the upper (nasal) airways in severe asthma, with anatomical compartmentalisation of the cytokine milieu.

Just three species dominate the airway microbiome in severe asthma, and their presence observed in both neutrophilic and non-neutrophilic phenotypes might explain the striking clinical efficacy of long-term, low dose azithromycin therapy in both phenotypes^23^, but most notably in those with greatest *H. influenzae* abundance, measured by qPCR^24^. In the BNA, symptom score (ACQ-7) was not linked to presence of a dominant pathogen, likely due to insensitivity of ACQ-7 to sputum volume or purulence. Indeed, azithromycin therapy produced a very small change in symptom score (0.1 points on ACQ-6) amongst severe asthmatics in the AMAZES trial^23^. Mechanistically, whilst azithromycin is primarily antibacterial, additional anti-viral^25^ and anti-inflammatory^26^ effects are reported. However, antimicrobial resistance with widespread azithromycin therapy is a significant concern^27^, and if the antibacterial effect is dominant, this could be addressed through targeting therapy to those with pathogen dominance identified through molecular techniques. We have previously reported that Nanopore sequencing identifies clinically-relevant airway pathogens and shows agreement with sputum culture and semi-quantitative pathogen specific qPCR^11^.

In this present study similar consistency is seen between a pathogen-dominant airway microbiome identified by Nanopore sequencing and positive pathogen specific qPCR results for *H. influenzae, S. pneumoniae, M. catarrhalis* and *P. aeruginosa* in samples from the Oxford cohort. The lower, 73% concordance between sequencing and qPCR results in Wessex samples (n=8/11) may be attributable to age-related sample degradation in this historical cohort, as total 16S load was >15-fold lower in Wessex than Oxford samples. A stringent qPCR cut-off of 1x10^6^ copies/ml was set for a positive result based on its ability to identify bacteria associated neutrophilic inflammation in COPD following longitudinal sampling and our previous report on agreement sputum culture results in asthma. It is worth noting that prevalence of potentially pathogenic microorganisms in COPD is relatively high^28^ and sputum culture lacks sensitivity^16^. If a lower threshold of 0.5x10^6^ copies/ml was set, concordance would have been 100%. Overall, Nanopore sequencing provided a detailed species level description of the overall microbiome. Currently, for operational reasons, qPCR on selected pathogens may offer the most accessible and accurate means of identifying pathogen-dominance for targeted therapy in routine clinical practice. However, rapid progress is being made with the operationalisation of Nanopore sequencing for direct clinical application, and a pilot study has shown successful implementation of Nanopore in a routine clinical setting with a turnaround from sample receipt to metagenomic report of < 7 hours, allowing same day reporting in >90% of cases^29^. This has important clinical implications as, with standardised workflows, more cost-effective sample multiplexing, and pipelines able to identify antimicrobial resistance genes, there is the potential to accurately guide antimicrobial therapies on a per-patient basis in severe asthma and other airways diseases.

Nanopore sequencing provides further insight into microbiome of distinct sputum inflammatory phenotypes. In addition to *H. influenzae, M. catarrhalis* and *S. pneumoniae, T. whipplei* was strongly associated with neutrophilic asthma. This predominantly intracellular bacterium causes chronic multi-system inflammation in Whipple’s disease and may be involved in airways disease. *T. whipplei* has been detected in up to 40% of severe asthmatics, linked to both eosinophilic^9^ and corticosteroid resistant inflammation^30^ albeit in smaller cohorts. Its ability to grow within acidic vacuoles, induce M2 macrophage polarisation and apoptosis are virulence traits capable of promoting chronic infection^31^. The asthmatic airway is susceptible to persistent infection with intracellular bacteria, most commonly non-typeable strains of *H. influenzae*^3^ (NTHi) which facultatively enter macrophages and airway epithelial cells, and is associated with airway neutrophilia and type 17 inflammatory mediators^32^. High dose ICS exposure may also render mucosa susceptible to *T. whipplei*. Further research is needed to determine if specific innate immune deficiencies promote this intracellular infection. The microbiome in eosinophilic asthma varies with disease severity and in severe disease displays high relative abundance of Firmicutes^17^. The Proteobacteria *M. catarrhalis* was associated with eosinophilic asthma in this study, alongside Firmicutes *S. intermedius* and *V. parvula*, though this comparison is underpowered and requires replication. BNA linked *M. catarrhalis* abundance to FeNO level, previously shown to correlate negatively with Proteobacteria abundance^17^. We observed a mutually exclusive relationship between high FeNO and abundance of *H. influenzae* suggesting that this bacterium may inhibit FeNO production by the airway mucosa.

In SA, notably in absence of a dominant pathogen, there is greater abundance of *Rothia* spp., previously detected in mild non-eosinophilic asthma^33^ and bronchiectasis, where abundance negatively correlated with IL-8, IL-1β, MMP-1, MMP-8 and MMP-9 in sputum^34^. Of these species, *R. mucilaginosa* is most often detected in chronic lung diseases and inhibits NF-κB activation in lung epithelium *in vitro*^34^. *R. mucilaginosa* is believed to produce anti-inflammatory mediators which directly inhibit NF-κB signalling, probably at the level of IKK activation and can reduce lung pathology *in vivo*^34^. It is not known why *Rothia* are more abundant in SA, but potentially as facultative anaerobes they may be better able to colonise airways with reduced oxygen tension secondary to mucus plugging. We saw association between IL-6 and *R. mucilaginosa*, but a strong negative correlation with FGF which itself positively correlated with oropharyngeal commensals. FGFs are a diverse group of growth factors exerting pleiotropic effects via tyrosine kinase receptors. FGF-2 promotes bronchial smooth muscle hyperplasia, shows higher expression in asthma, and with FGF-1 stimulates MAPK-dependent VEGF production, inhibited by azithromycin and dexamethasone^35^. Thus, microbial composition could influence airway remodelling processes further manipulated by corticosteroids and macrolides.

Integration of metagenome and inflammatory mediators, together with BNA offer additional novel insights. Overall, a pattern emerges linking a pathogen-dominant microbiome and type-1 inflammation (IL-1β, TNF) with neutrophilic infiltration (IL-8, YKL40), release of neutrophil granule proteins and proteases (MMP8, MMP9, MPO and elastase), counter regulatory mechanisms (TIMP-1, IL-10), and macrophage recruitment (MIP1α and MIP1β) which could be facilitated through IL-6 signalling (IL-6SR). *H. influenzae* correlated with the anti-inflammatory cytokine IL-10, consistent with data in *in vitro*^36^ and in COPD^37^. Co-production of IL-10/TNF, induced via MyD88-dependent TLR activation following NTHi infection, may offer a regulatory response, and *H. influenzae* could harness excess IL-10 secretion as a virulence strategy. This could be explored further in murine studies.

*H. influenzae* also correlated strongly with sputum ECP (RNase 3), a cytotoxic eosinophil granule basic protein released on eosinophil activation. ECP has antibacterial properties but is also associated with airways remodelling, and inflammation and can induce mucus hypersecretion, potentially explaining its association with chronic bronchitis^38^. The strong association we observe could result either from eosinophilic inflammation predisposing to acquisition of *Haemophilus*, or, perhaps more likely, is a harmful consequence of *Haemophilus* infection. To this end data from recent on-biologic studies are suggestive. In the MEX study respiratory bacteria were detectable at high copy number at stable state in 36% of patients established on mepolizumab, a higher proportion than our cohort^39^. Furthermore therapy with benralizumab – which causes more profound eosinophil depletion, besides effects on other IL-5R-expressing cell types – is associated with a significant threefold increased incidence of respiratory infections, consistent with an antibacterial role of eosinophils, though the situation is likely complex due to the opposing effects of eosinophil and bacteria-induced mucus plugging^40^.

IL-1RA and TIMP-1 were differentially regulated in presence of a dominant pathogen. IL-1RA, an antagonist to IL-1β and its receptor, was reduced, consistent with reduced IL-1RA:IL-1β ratio in neutrophilic asthma^41^, potentially representing IL-1β quenching during infection. IL-1RA is upregulated in steroid-naïve atopic asthma^42^, with IL-1RA polymorphisms increasing asthma risk. ICS dampens IL-1RA production, as supported by relatively higher concentrations in healthy/mild asthmatic sputum (Figure 2A). TIMP-1, also elevated in presence of a dominant pathogen, possesses antiprotease activity, and MMP-9:TIMP-1 ratio is reduced in asthma and chronic bronchitis^43^, correlating inversely with airflow obstruction, with TIMP-1 polymorphisms associated with female asthma^44^. Thus, whilst TIMP-1 blocks proteolytic activity, in excess it might reduce extracellular matrix turnover resulting in remodelling. IL-10, IL-1RA and TIMP-1 are thereby integral to complex regulatory networks with likely differing roles dependant on the wider inflammatory landscape. This could be better characterised through dedicated transcriptomic and proteomic studies in well phenotyped severe asthmatics.

We have shown that the ‘one airway, one disease’ concept does not apply to the airway microbiome in severe asthma where distinct microbial niches are maintained in the nasopharynx and lower airway. This is consistent with mild atopic asthma and health^7,22^. The nasal microbiome can be informative and has been shown to predict exacerbation risk in paediatric asthma when dominated by *Moraxella* species, but our data and previous studies in mild asthma suggests nasopharyngeal samples should not replace lower airway sampling in stable state or exacerbation in adult asthma^45^. This distinction between paediatric and adult asthma remains unexplained. We found the cytokines IL-20^46^ and IL-2^47^ in sputum, which have been linked to airway remodelling and allergic inflammation in asthma respectively, to correlate with nasal proteins involved in diverse cellular responses. The importance of cross-signalling between these compartments is not known but of interest given the burden of comorbid sinonasal disease in severe asthma.

This study had some limitations. Samples from the Wessex cohort, collected between 2009 and 2014, are from a pre-biologic era with widespread maintenance oral corticosteroid use which may confound detection of sputum inflammatory mediators. Sample collection and storage protocols can affect quality of metagenomic data, particularly in low biomass samples, and we suspect some storage-related DNA degradation in Wessex samples as DNA was extracted after <11½ years of storage. This study has limited power given the moderate sample size, particularly the low number of healthy controls and those with mild asthma, which will limit detection of significant but smaller differences in microbial composition within the cohort. Future studies could be strengthened by including a larger cohort with mild and moderate asthma, and longitudinal data spanning exacerbations. Due to the nature of human respiratory samples with high host DNA content^11,48^, sequencing depth in direct from clinical sample extracts can be inherently limited and variable, despite efforts to deplete host DNA. Previous work by Sanderson *et al*. has shown that ~20x coverage is required for consensus whole genome sequences allowing confident identification of antibiotic resistant determinants, and SNPs, or similarly strain inferences. Consistent high depth sequences of this order are typically derived following sequencing of bacterial isolates; clinical samples show far greater variation. The sequencing depth in this study was therefore inadequate for such functional analyses in the full dataset. Approached to overcome these limitations could include achieving greater depth by sequencing fewer samples per flow cell or using probe-based enrichment of pathogen specific DNA enabling deeper sequencing.

In conclusion, we show that a pathogen-dominant microbiome is a common treatable trait in severe asthma, present in 20-30% of patients, most often with *H. influenzae*. It is accompanied by neutrophilic inflammation, elevated airway proteases and disruption of counterregulatory immune responses which could facilitate pathogen persistence and airway remodelling.

## Supplementary Material

Online supplement

Supplementary table

## Figures and Tables

**Table 1:** Clinical characteristics of the participants

Characteristic	Wessex				Oxford
	Healthy (n=5)	Mild Asthma (n=10)	Severe Asthma (n=51)		Severe asthma (n=30)
**Male sex**, n (%)	3 (60)	5 (50)	22 (43)		14 (46.7)
**Age (years)**, Mean (SD)	29 (12.3)	44.1 (15.9)	49.1 (14.7)		59.5 (15.0)
**BMI (kg/m**^2^), Mean (SD)	25 (4.4)	27.5 (6.0)	32.2 (8.1)		29.2 (6.2)
**Presence of atopy**, n (%)	0 (0)	5 (50)	35 (69)		6 (20.0)
**Presence of nasal polyps**, n (%)	0 (0)	0 (0)	6 (12)		10 (33.3)
**Smoking status**, n (%)					
Never	4 (80)	5 (50)	27 (53)		22 (73.3)
Current	0 (0)	1 (10)	17 (33)		1 (3.3)
Ex-smoker	1 (20)	4 (40)	7 (14)		7 (23.4)
**Pack years**, Mean (SD)	7.5 -	1 (1.2)	8.4 (13.0)		12 (10)
**Baseline inhaled corticosteroid use (BDP** **eq., mcg/d)**, Median (Q1,Q3)	na na	300 (0, 400)	2080 (1800, 3575)		2000 (1600, 2000)
**Maintenance oral corticosteroid**, n (%)	na na	0 (0)	19 (37)		4 (13.3)
**Biologic therapy**, n (%) (mepolizumab)	0 (0)	0 (0)	0 (0)		3 (10)
**Unscheduled GP/ hospital visits in 12** **months**, Median (Q1,Q3)	na na	0 (0, 0)	3 (0, 7)		2 (0,6)
**FEV1 (%predicted)**, Mean (SD)	96 (9.8)	107 (12.8)	73.6 (24.5)		73.8 (20.6)
**FEV1/FVC**, Mean (SD)	1 (0.2)	0.8 (0.2)	0.6 (0.1)		0.6 (0.1)
**FeNO (ppb)**, Median (Q1,Q3)	11 (8, 11)	31 (16, 45)	10 (7, 17)		38 (18, 64)
**Blood eosinophils (x10^9^/L)**, Median (Q1,Q2)	nd nd	0.4 (0.2, 0.4)	0.2 (0.1, 0.3)		0.4 (0.2, 0.7)
**Sputum eosinophils (%)**, Median (Q1,Q3)	1.1 (0.9, 3.3)	2.2 (1.2, 3.1)	1 (0.3, 7.7)		4.5 (1.0, 42.2)
**Sputum neutrophils (%)**, Median (Q1,Q3)	31 (20.2, 37.3)	23.9 (16.7, 31.7)	51.8 (28.5, 76.1)		38.1 (17.0, 86.0)
**Sputum inflammatory phenotype**, n (%)					
Eosinophilic	1 (20)	1 (10)	13 (25.5)		11 (36.7)
Neutrophilic	0 (0)	0 (0)	13 (25.5)		6 (20.0)
Mixed granulocytic	0 (0)	0 (0)	3 (6)		1 (3.3)
Paucigranulocytic	2 (40)	7 (70)	20 (39)		4 (13.3)
No data	2 (40)	2 (20)	2 (4)		8 (26.7)

BMI, body mass index; FEV1, Forced expiratory volume in 1s; FVC, Forced vital capacity; FeNO, exhaled nitric oxide; Inflammatory phenotypes: eosinophilic ≥3% sputum eosinophils, neutrophilic ≥61% sputum neutrophils and <3% eosinophils, mixed granulocytic ≥61% sputum neutrophils and ≥3% eosinophils, paucigranulocytic <61% sputum neutrophils and <3% eosinophils; SD, standard deviation; Q1, quartile 1; Q3, quartile3; na, not applicable; nd, no data

## Data Availability

Raw FASTQ data are available on the European Nucleotide Archive, project accession PRJEB62780.
