# Supplementary material for: Species-level, metagenomic and proteomic analysis of microbe-immune interactions in severe asthma
Source: Allergy. Author manuscript; Available in PMC 2025 Jun 21. (PMC7617782; doi:10.1111/all.16269)

**Supplementary and Results:** Species-level, metagenomic and proteomic analysis of microbe-immune interactions in severe asthma

Table of Contents

[Supplementary Methods 2](#_Toc166743701)

[1. Microbial DNA extraction with human DNA depletion 2](#_Toc166743702)

[2. Oxford Nanopore Technologies (ONT) sequencing 2](#_Toc166743703)

[3. Pathogen specific PCR analysis 2](#_Toc166743704)

[4. Measurement of inflammatory mediators 2](#_Toc166743705)

[5. Bioinformatic analysis 3](#_Toc166743706)

[References 5](#_Toc166743707)

[Supplementary Results 6](#_Toc166743708)

[Table E1 Clinical characteristics of severe asthma in Wessex Severe Asthma Cohort 7](#_Toc166743709)

[Table E2 Clinical characteristics of severe asthma in Oxford Severe Asthma Cohort 8](#_Toc166743710)

[Figure E1 Study design 9](#_Toc166743711)

[Figure E2 Heatmap of relative abundance of species 10](#_Toc166743712)

[Figure E3 PCA of metagenomic profiles 12](#_Toc166743713)

[Figure E4 Taxonomic profiles in sputum in severe asthma 13](#_Toc166743714)

[Table E3 Top species in health 16](#_Toc166743715)

[Table E4 Top species in mild asthma 17](#_Toc166743716)

[Table E5 Top species in severe asthma 18](#_Toc166743717)

[Table E6 Single pathogen dominance in severe asthma 19](#_Toc166743718)

[Table E7 Species by inflammatory phenotype 20](#_Toc166743719)

[Figure E5 Sankey visualisations in Wessex Cohort 21](#_Toc166743720)

[Figure E6 Proteobacteria:firmicute ratios 22](#_Toc166743721)

[Table E8 Sputum cytokines by presence of infection 23](#_Toc166743722)

[Table E9 Bayesian Network Analysis variables 24](#_Toc166743723)

[Figure E7 Correlations between inflammatory mediators 25](#_Toc166743724)

[Figure E8 Relationship of FeNO to *H. influenzae* abundance 26](#_Toc166743725)

[Figure E9 Pairwise associations between bacterial species 27](#_Toc166743726)

[Figure E10 Upper and lower airway microbiome and cytokines 28](#_Toc166743727)

[Figure E11 PCA of metagenomic profiles 29](#_Toc166743728)

# **Supplementary Methods**

## **Microbial DNA extraction with human DNA depletion**

Established methods were used for microbial DNA extraction^1^. Briefly, homogenised sputum or nasal lavage underwent differential centrifugation prior to DNA extraction using the QIAamp DNA Mini Kit (Qiagen, Manchester, UK) as per manufacturer’s instructions with previously described modification^1^.

## **Oxford Nanopore Technologies (ONT) sequencing**

Libraries were prepared for sequencing on an Oxford Nanopore GridION (Oxford Nanopore Technologies (ONT), Oxford, UK) using the Rapid PCR Barcoding kit (SQK-RPB004) with modifications to the manufacturer’s protocol as previously reported ^1^. Samples were sequenced on ONT FLO-MIN106D (ONT v.R9.4.1) flow cells in batches of 6 samples per flow cell.

## **Pathogen specific PCR analysis**

Quantitative real-time PCR (qPCR) was performed to quantify relative abundance of *H. influenzae*, *S. pneumoniae*, *S. aureus*, *M. catarrhalis* and *P. aeruginosa* using methods and reactions conditions previously described^1^.

## **Measurement of inflammatory mediators**

Existing dataset utilised from Hinks *et al*.^2^. Inflammatory mediators measured from sputum by enzyme-linked immunosorbent assays (ELISA) were myeloperoxidase (MPO), elastase (Hycult), eosinophil cationic protein (MBL International), interleukin (IL)-5 (Abnova), IL-6, ENA-78, Eotaxin, FGF, osteopontin, VEGF, YKL-40 (R&D Systems), tryptase and α2-macroglobulin (A Walls4), as per manufacturer’s instructions. Fluoroenzyme immunoassay was used for total and specific IgE (ImmunoCAP, Phadia) and cytokine bead array (Luminex®) for matrix metalloproteases (MMPs), tissue inhibitor of matrix metalloproteinase 1 (TIMP-1), G-CSF, GM-CSF, Gro-α, IFN-γ, IL-1 α, IL-1 β, IL-1RA, IL-2, IL-4, IL-8, IL6SR, IL-10, IL-13, IL-17, MCP-1, MIP-1 α, MIP-1 β and TNF-α.

In addition, sputum and nasal lavage samples were analysed by O-link (Uppsala, Sweden) using a 92 mediator in-house Proseek Multiplex Inflammation I panel including: 4E-BP1, Adenosine deaminase, Artemin, Axin-1, Beta-nerve growth factor, Caspase-8, C-C motif chemokine 11, C-C motif chemokine 13, C-C motif chemokine 19, C-C motif chemokine 2, C-C motif chemokine 20, C-C motif chemokine 23, C-C motif chemokine 25, C-C motif chemokine 28, C-C motif chemokine 3, C-C motif chemokine 4, C-C motif chemokine 7, C-C motif chemokine 8, CD antigen: CD, CD antigen: CD137, CD antigen: CD150, CD antigen: CD318, CD antigen: CD40, CD antigen: CD6, CD antigen: CD8a, CSF-1, C-X-C motif chemokine 1, C-X-C motif chemokine 10, C-X-C motif chemokine 11, C-X-C motif chemokine 5, C-X-C motif chemokine 6, C-X-C motif chemokine 9, Cystatin-D, Delta and Notch-like epidermal growth factor-related receptor, FGF-19, FGF-21, FGF-23, FGF-5, Flt3 ligand, Fractalkine, Hepatocyte growth factor, hGDNF, HVEM-L, IL-10R subunit alpha, IL-10R subunit beta, IL-12B, IL-15R-alpha, IL-20R-alpha, IL-22R-alpha-1, IL-2R subunit beta, Interferon gamma, Interleukin-1 alpha, Interleukin-10, Interleukin-13, Interleukin-17A, Interleukin-17C, Interleukin-18, Interleukin-18 receptor 1, Interleukin-2, Interleukin-20, Interleukin-24, Interleukin-33, Interleukin-4, Interleukin-5, Interleukin-6, Interleukin-7, Interleukin-8, Kit ligand, LAP, LIF, LIF-R, Lymphotoxin-alpha, MMP-1, MMP-10, NAIL, Neurotrophin-3, Neurturin, Oncostatin-M, Osteoprotegerin, PD-L1, Protein S100-A12, RANKL soluble, SIR2-like protein 2, STAM-binding protein, Sulfotransferase 1A1, TGF-alpha, Thymic stromal lymphopoietin, TRAIL, Tumor necrosis factor, TWEAK, U-plasminogen activator, VEGF-A, 4E-BP1, Adenosine deaminase, Artemin, Axin-1, Beta-nerve growth factor, Caspase-8, C-C motif chemokine 11, C-C motif chemokine 13, C-C motif chemokine 19, C-C motif chemokine 2, C-C motif chemokine 20, C-C motif chemokine 23, C-C motif chemokine 25, C-C motif chemokine 28, C-C motif chemokine 3, C-C motif chemokine 4, C-C motif chemokine 7 and C-C motif chemokine 8.

## **Bioinformatic analysis**

Sequences were basecalled using Guppy (ONT, version 3.3.0+ef22818), analysed using the CRuMPIT pipeline, and taxonomic classification of trimmed reads performed using Centrifuge as previously described^1^. Kraken style reports, produced following taxonomic classification of trimmed reads, were used for downstream analysis. A description of bioinformatics and secondary analysis is provided in the GitLab repository: <https://gitlab.com/ModernisingMedicalMicrobiology/wessex_analysis>

Metagenomic and integrated cytokine analysis;

5.1 Visualisation of metagenomic data; Kraken style reports were produced following taxonomic classification of trimmed reads. Raw species level reads (Wessex, n=66; Oxford, n=30) from sputum samples were combined into two datasets, 7644 and 3860 unique species were identified respectively. Low abundance species (<1000 reads across all samples) were removed prior to normalisation (DESeq2) thereby reducing unique species to 253 and 72, respectively. The two datasets were merged by common detected species (n=70) and z-scores derived for heatmaps. Principal component analysis (PCA) plots were produced to provide a low dimensional representation of the 3 participant groups (healthy, moderate and severe asthma) in the Wessex cohort and the two severe asthma groups (Wessex and Oxford). Groups of samples were compared by permutational multivariate ANOVA (PERMANOVA) performed using the adonis function in the Vegan package. Wald test used to identify species uniquely abundant or depleted in HC, MA and SA, or by inflammatory phenotype (using DESeq2, log fold change [LFC] > 1 or <1 respectively, adjusted P < 0.05).

5.2 Sankey visualisations*;* Individual or combined Kraken style reports used to produce Sankey visualisations of taxonomic profiles using Pavian^3^. Where a dominant pathogen was identified on metagenomic sequencing in more than one individual, the relative abundance of these bacteria (*H. influenzae, M. catarrhalis, S. pneumonia*) was compared against the relative abundance of other detected species within each sample using Wilcoxon rank-sum test, using Benjamini-Hochberg [BH] correction FDR 0.05, in order to provide statistical support for the skewed metagenomic profiles shown.

5.3 Normalisation, integration and visualisation of cytokine and metagenomic data*;* Raw cytokine measurements were centralised by z-score, clustering was performed by cytokine and subjects, with clustering distances visualised in heatmap representing correlation distances. To integrate metagenomic and cytokine data within the combined Wessex dataset (HC, MA, SA), species level reads were normalised, and log transformed, while cytokines were centralised by z-score prior to computation of Spearman correlation between shared species (~253) and cytokines by matching across samples (adjusted for multiple comparisons with Benjamini–Hochberg procedure [FDR 0.05]). The 50 species with greatest variance in abundance across subjects were selected for clustering analysis by cytokines (columns) and species (rows).

5.4 Bayesian network analysis*;* Connections between cytokines associated to airways infection, clinical parameters, and differentially abundant bacterial species in severe asthma was explored by Bayesian network analysis (Genie 2.0; Decision Systems Laboratory, University of Pittsburgh, Pittsburgh, Pa). Data from 51 severe asthmatics (60 variables) were discretised into 2 (binary variables) or 3 to 5 (continuous variables) bins (variables shown in Table S8). Using the ‘clustering algorithm’^4^ 34/60 variables were retained in the model. The Bayesian network generated was re-plotted using shinyBN^5^. In a subsequent separate analysis strengths of associations were further explored using Pearson (between species) or Spearman correlation (between cytokines). Positive linear correlations are highlighted with a green edge.

5.5 Integration of nasal lavage and sputum metagenomics and O-link data*;* Low abundance species (<200 reads across all individuals) were removed from nasal lavage and sputum samples (filtering to 94 and 349 species respectively) prior to scaling bacterial reads within samples. A combined dataset of shared nasal lavage and sputum species was produced (n=23) by selecting organism with highest variance in abundance across all samples (P<0.05). A PCA plot was produced using these samples and the two groups of samples compared by permutational multivariate ANOVA (PERMANOVA) performed using the adonis function in the Vegan package. Spearman correlation was performed using paired O-link proteomic data (92 protein panel) in sputum and nasal lavage and significantly correlated pairs of cytokines were filtered (P<<0.001) following adjustment for multiple corrections (n=38; sputum = 16, nasal=22).

## **References**

1. Jabeen, M.F.*, et al.* Identifying Bacterial Airways Infection in Stable Severe Asthma Using Oxford Nanopore Sequencing Technologies. *Microbiol Spectr* **10**, e0227921 (2022).
2. Hinks, T.S.C.*, et al.* Multidimensional endotyping in patients with severe asthma reveals inflammatory heterogeneity in matrix metalloproteinases and chitinase 3-like protein 1. *J Allergy Clin Immunol* **138**, 61-75 (2016).
3. Breitwieser, F.P. & Salzberg, S.L. Pavian: interactive analysis of metagenomics data for microbiome studies and pathogen identification. *Bioinformatics* **36**, 1303-1304 (2020).
4. BayesFusion. GeNIe Clustering algorithm. https://support.bayesfusion.com/docs/GeNIe/algorithms_clustering.html (accessed 2023-11-27.
5. Chen, J.*, et al.* shinyBN: an online application for interactive Bayesian network inference and visualization. *BMC Bioinformatics* **20**, 711 (2019).

# **Supplementary Results**

**Data sharing**

Raw FASTQ data are available on the European Nucleotide Archive with the project accession PRJEB62780. Remaining data analysed and presented in this study are available from the corresponding author on reasonable request, providing the request meets local ethical and research governance criteria after publication. Patient-level data will be anonymised.

## **Table E1 Clinical characteristics of severe asthma in Wessex Severe Asthma Cohort**

Clinical characteristics of participants with severe asthma from the Wessex Severe Asthma Cohort without or with airways infection; BMI, body mass index; FEV1, Forced expiratory volume in 1s; FVC, Forced vital capacity; FeNO, exhaled nitric oxide; Inflammatory phenotypes: eosinophilic ≥3% sputum eosinophils, neutrophilic ≥61% sputum neutrophils and <3% eosinophils, mixed granulocytic ≥61% sputum neutrophils and ≥3% eosinophils, paucigranulocytic <61% sputum neutrophils and <3% eosinophils; SD, standard deviation; Q1, quartile 1; Q3, quartile3. *Student's t test, ^†^Mann-Whitney U test, ^‡^Fisher exact test

|  |  |  |  |  |  |  |
| --- | --- | --- | --- | --- | --- | --- |
| **Characteristic** | **Airways infection absent** (n=41) | | **Airways infection present** (n=11) | | **P value** |  |
| **Male gender**, n (%) | 16 | (39.0) | 6 | (54.5) | 0.49§ |  |
| **Age (years)**, Mean (SD) | 49.0 | (14.3) | 51.0 | (10.2) | 0.63† |  |
| **BMI (kg‎/m^2^)**, Mean (SD) | 32.9 | (8.0) | 29.1 | (8.4) | 0.20† |  |
| **Presence of atopy**, n (%) | 25 | (54.5) | 7 | (63.6) | >0.99§ |  |
| **Presence of nasal polyps**, n (%) | 6 | (14.6) | 1 | (9.0) | >0.99§ |  |
| **Smoking status**, n (%) |  |  |  |  |  |  |
| Never | 22 | (53.6) | 5 | (45.5) | 0.74§ |  |
| Current | 4 | (9.8) | 2 | (18.2) | 0.60§ |  |
| Ex-smoker | 15 | (36.6) | 4 | (36.3) | >0.99§ |  |
| **Pack years**, Mean (SD) | 8.0 | (12.8) | 9.5 | (14.2) | 0.73† |  |
| **Baseline inhaled corticosteroid use (BDP eq., mcg/d)**, Median (Q1,Q3) | 2080 | (1680, 3500) | 2000 | (1500,2600) | 0.44‡ |  |
| **Maintenance oral corticosteroid**, n (%) | 16 | (39.0) | 3 | (27.3) | 0.47§ |  |
| **Unscheduled GP/ hospital visits in 12 months**, Median (Q1,Q3) | 1 | (0,4) | 1 | (0,3) | 0.98‡ |  |
| **FEV1 (%predicted)**, Mean (SD) | 65.5 | (25.0) | 61.4 | (27.8) | 0.61† |  |
| **FEV1/FVC**, Mean (SD) | 0.6 | (0.1) | 0.6 | (0.2) | 0.17† |  |
| **FeNO (ppb)**, Mean (SD) | 26.0 | (29) | 29.0 | (26) | 0.60† |  |
| **Blood eosinophils (x10^9^/L)**, Median (Q1,Q2) | 0.2 | (0.1,0.3) | 0.2 | (0.1, 0.6) | 0.67‡ |  |
| **Sputum eosinophils (%)**, Median (Q1,Q3) | 1.0 | (0.3, 5.5) | 1.5 | (0.9,10.5) | 0.21‡ |  |
| **Sputum neutrophils (%)**, Median (Q1,Q3) | 46.8 | (19.9, 62.5) | 78 | (48.8,87.5) | **0.01‡** |  |
| **Sputum inflammatory phenotype**, n (%) |  |  |  |  |  |  |
| Eosinophilic | 9 | (22.0) | 4 | (36.0) | 0.44§ |  |
| Neutrophilic | 8 | (19.0) | 5 | (45.0) | 0.07§ |  |
| Mixed granulocytic | 2 | (5.0) | 1 | (9.5) | >0.99§ |  |
| Paucigranulocytic | 20 | (49.0) | 1 | (9.5) | 0.11§ |  |
| No data | 2 | (5.0) | 0 | (0.0) |  |  |
| BMI=body mass index; FEV1=Forced expiratory volume in 1s; FVC=Forced vital capacity; FeNO=exhaled nitric oxide; Inflammatory phenotypes: eosinophilic ≥3% sputum eosinophils, neutrophilic ≥61% sputum neutrophils and <3% eosinophils, mixed granulocytic ≥61% sputum neutrophils and ≥3% eosinophils, paucigranulocytic <61% sputum neutrophils and <3% eosinophils; SD= standard deviation, Q1=quartile 1, Q3=quartile3. †Student's t test, ‡Mann-Whitney U test, §Fisher exact test | | | | | |  |
|  |  |  |  |  |  |  |
|  |  |  |  |  |  |  |

## **Table E2 Clinical characteristics of severe asthma in Oxford Severe Asthma Cohort**

Clinical characteristics of participants with severe asthma from the Oxford Severe Asthma Cohort without or with airways infection; BMI, body mass index; FEV1, Forced expiratory volume in 1s; FVC, Forced vital capacity; FeNO, exhaled nitric oxide; Inflammatory phenotypes: eosinophilic ≥3% sputum eosinophils, neutrophilic ≥61% sputum neutrophils and <3% eosinophils, mixed granulocytic ≥61% sputum neutrophils and ≥3% eosinophils, paucigranulocytic <61% sputum neutrophils and <3% eosinophils; SD, standard deviation; Q1, quartile 1; Q3, quartile3. *Student's t test, ^†^Mann-Whitney U test, ^‡^Fisher exact test

| **Characteristic** | **Airways infection absent** (n=22) | | **Airways infection present** (n=8) | | **P value** |
| --- | --- | --- | --- | --- | --- |
| **Male gender**, n (%) | 11 | (50) | 3 | (37.5) | 0.69§ |
| **Age (years)**, Mean (SD) | 58.0 | (13.7) | 66.0 | (12.1) | 0.15† |
| **BMI (kg‎/m^2^)**, Mean (SD) | 29.0 | (6.0) | 30.0 | (6.6) | 0.72† |
| **Presence of atopy**, n (%) | 5 | (22.7) | 1 | (4.5) | >0.99§ |
| **Presence of nasal polyps**, n (%) | 7 | (31.8) | 1 | (4.5) | 0.39§ |
| **Smoking status**, n (%) |  |  |  |  |  |
| Never | 16 | (72.7) | 6 | (75.0) | >0.99§ |
| Current | 0 | (0.0) | 1 | (4.5) | 0.27§ |
| Ex-smoker | 6 | (27.3) | 1 | (4.5) | 0.63§ |
| **Pack years**, Mean (SD) | 7.1 | (3.6) | 25.5 | (9.2) | 0.01† |
| **Baseline inhaled corticosteroid use (BDP eq., mcg/d)**, Median (Q1,Q3) | 2000 | (1600, 2000) | 2000 | (1700, 2000) | 0.86‡ |
| **Maintenance oral corticosteroid**, n (%) | 3 | (13.6) | 1 | (4.5) | 0.94§ |
| **Unscheduled GP/ hospital visits in 12 months**, Median (Q1,Q3) | 2 | (0, 7) | 2 | (0, 12) | 0.98‡ |
| **FEV1 (%predicted)**, Mean (SD) | 75.0 | (21.5) | 61.8 | (30.0) | 0.19† |
| **FEV1/FVC**, Mean (SD) | 0.6 | (0.1) | 0.7 | (0.1) | 0.81† |
| **FeNO (ppb)**, Mean (SD) | 73.0 | (73.2) | 29.0 | (16.1) | 0.11† |
| **Blood eosinophils (x10^9^/L)**, Median (Q1,Q2) | 0.4 | (0.2, 0.9) | 0.3 | (0.1, 0.5) | 0.53‡ |
| **Sputum eosinophils (%)**, Median (Q1,Q3) | 12.0 | (1.3, 51.5) | 1.0 | (0.1, 1.6) | **0.02‡** |
| **Sputum neutrophils (%)**, Median (Q1,Q3) | 31.0 | (12.8, 56.4) | 90.5 | (51.5, 98.6) | **0.03‡** |
| **Sputum inflammatory phenotype**, n (%) |  |  |  |  |  |
| Eosinophilic | 11 | (50.0) | 0 | (0.0) | **0.01§** |
| Neutrophilic | 2 | (9.0) | 4 | (50.0) | **0.03§** |
| Mixed granulocytic | 1 | (4.5) | 0 | (0.0) | >0.99§ |
| Paucigranulocytic | 3 | (13.6) | 1 | (4.5) | >0.99§ |
| No data | 5 | (22.7) | 3 | (37.5) |  |
| BMI=body mass index; FEV1=Forced expiratory volume in 1s; FVC=Forced vital capacity; FeNO=exhaled nitric oxide; Inflammatory phenotypes: eosinophilic ≥3% sputum eosinophils, neutrophilic ≥61% sputum neutrophils and <3% eosinophils, mixed granulocytic ≥61% sputum neutrophils and ≥3% eosinophils, paucigranulocytic <61% sputum neutrophils and <3% eosinophils; SD= standard deviation, Q1=quartile 1, Q3=quartile3. *Student's t test, ‡Mann-Whitney U test, §Fisher exact test | | | | | |

## **Figure E1 Study design**

(Created with BioRender.com)


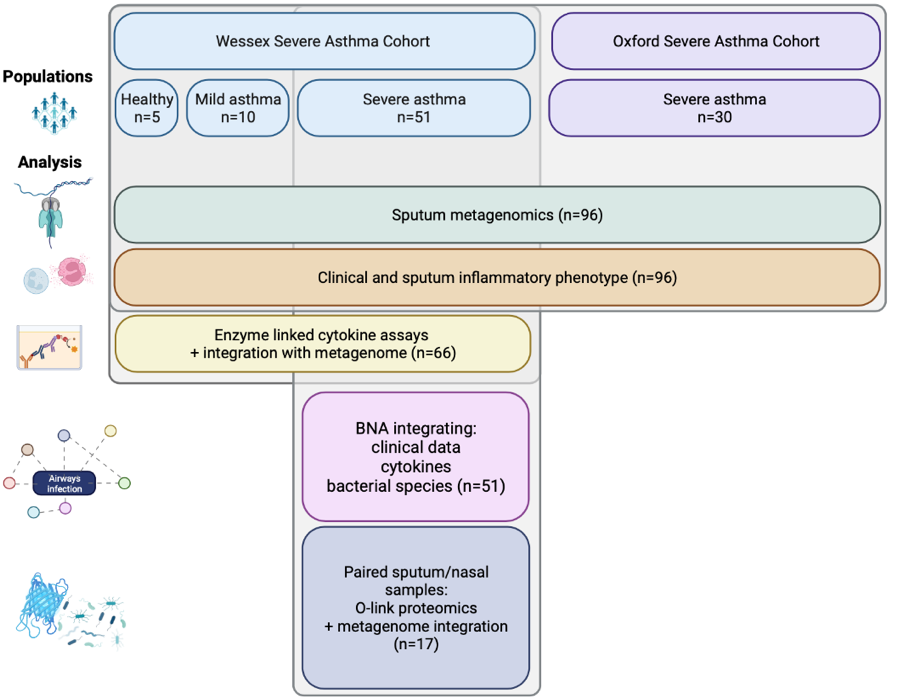


## **Figure E2 Heatmap of relative abundance of species**

Heatmap of relative abundance of species from induced sputum sequenced samples using ONT; Z-scores, denoted by shade, represent the number of standard deviations above the mean number of reads per taxon for each sample. Cohorts include (a) Wessex and (b) Oxford. Clustering performed independently on samples and most differentially abundant bacterial species. Sputum inflammatory phenotypes are eosinophilic (E), neutrophilic (N), mixed granulocytic (M) and paucigranulocytic (P). Positive pathogen specific PCR result (>1x10^6^copies/ml) is indicated in black: S. aureus (Saur), P. aeruginosa (PsA), S. pneumoniae (Spneu), M. catarrhalis (Mcat) and H. influenzae (Hinf). Total eubacteria (16S) PCR load is denoted by shade (x10^6^copies/ml).


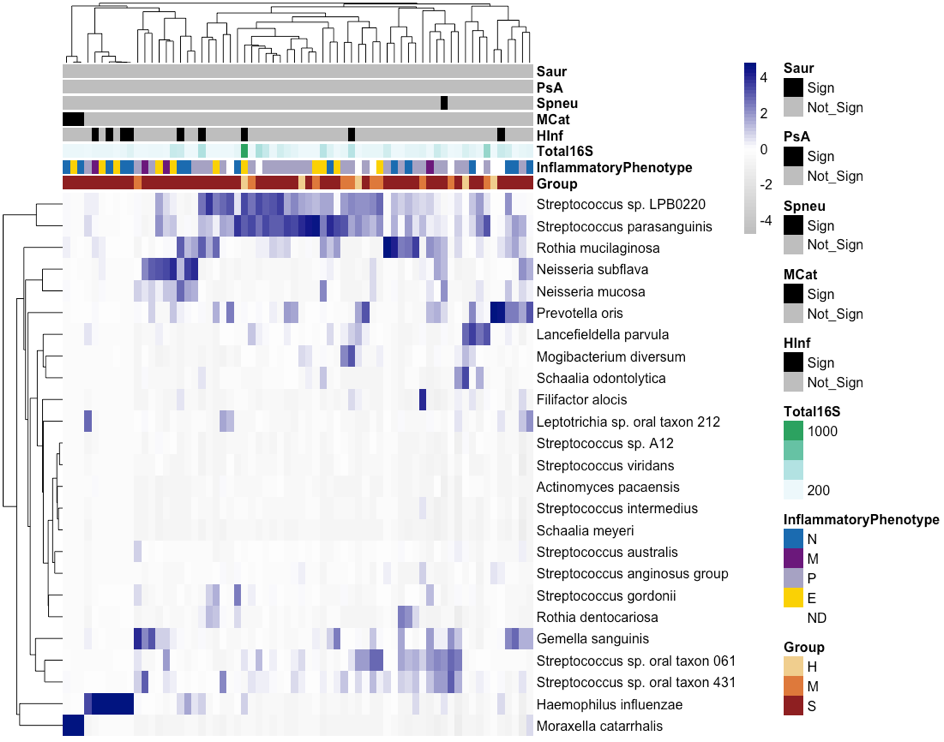


(a)


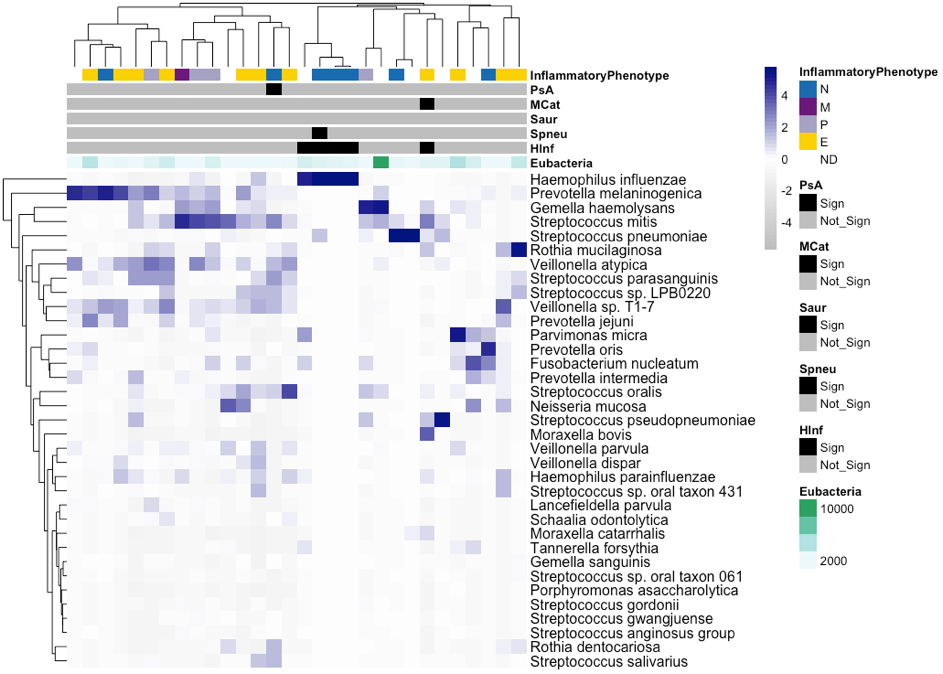


(b)

## **Figure E3 PCA of metagenomic profiles**

Principal component analysis (PCA) based on metagenomic profiles with convex hulls plotted for (a) Wessex severe asthma cohort, representing healthy (H), moderate asthma (M), severe asthma (S) and severe asthma with presence of dominant pathogen (S+D), (PERMANOVA = 0.001); and (b) severe asthmatics from the Oxford (OX) and Wessex (WES) cohorts (PERMANOVA = 0.19). First two principal components are shown.

A)


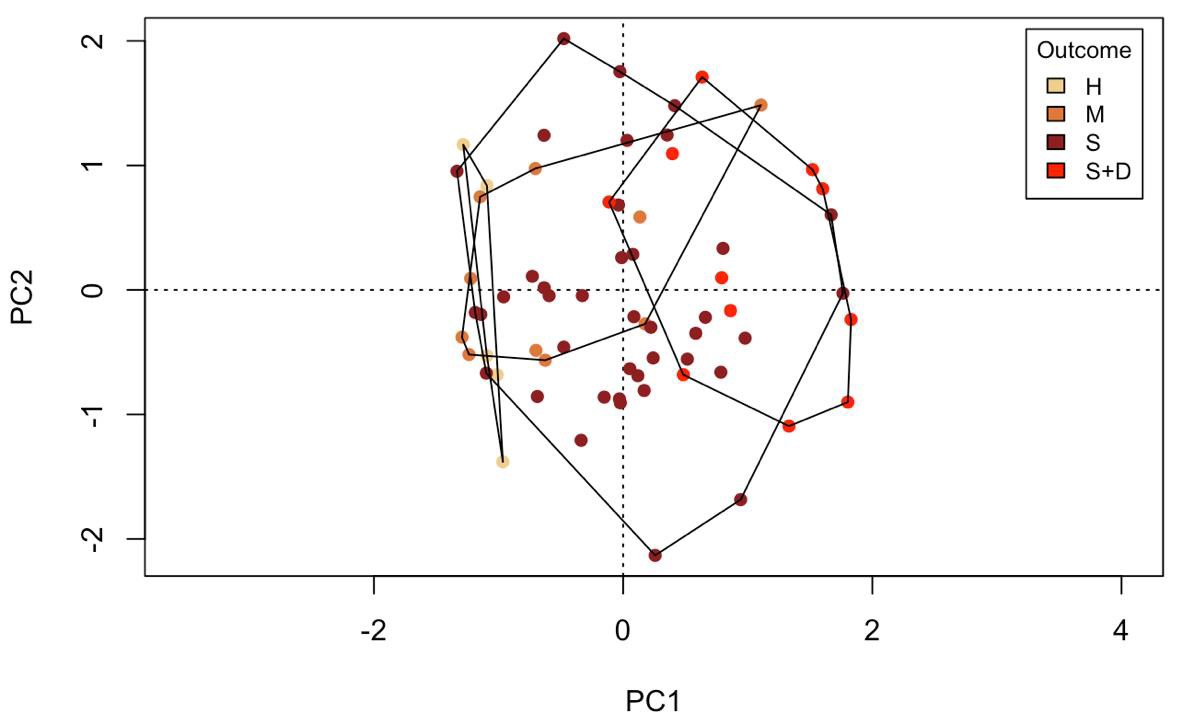


B)


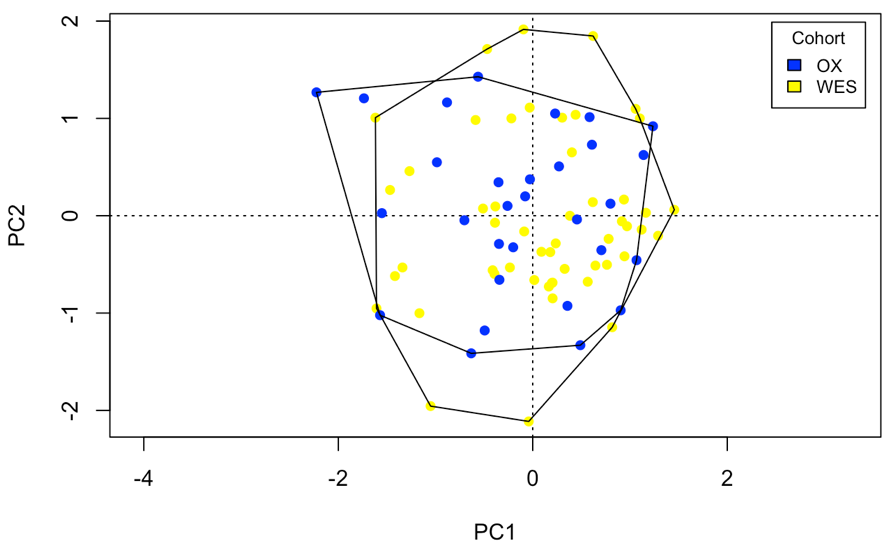


## **Figure E4 Taxonomic profiles in sputum in severe asthma**

Sankey visualisation of taxonomic profiles in severe asthma in the absence or presence of a single dominant pathogen using Pavian; Kraken reports combined from patients with no single dominant organism on metagenomic sequencing from (A) Wessex (n=40) and (E) Oxford (n=22) cohorts for visualisation. Skewed taxonomic in the presence of a single dominant pathogen profiles (Wilcoxon rank-sum test, Benjamini-Hochberg [BH] correction FDR 0.05; P≤0.01), one of (B)+(F) H. influenzae ([B] Wessex, n= 6; [F] Oxford, n=4), (C) M. catarrhalis (Wessex, n=3) or S. pneumoniae (Wessex, n=2) have been generated using the same method. (G) S. pneumoniae (Oxford, n=2), (H) P. aeruginosa (Oxford, n=1).


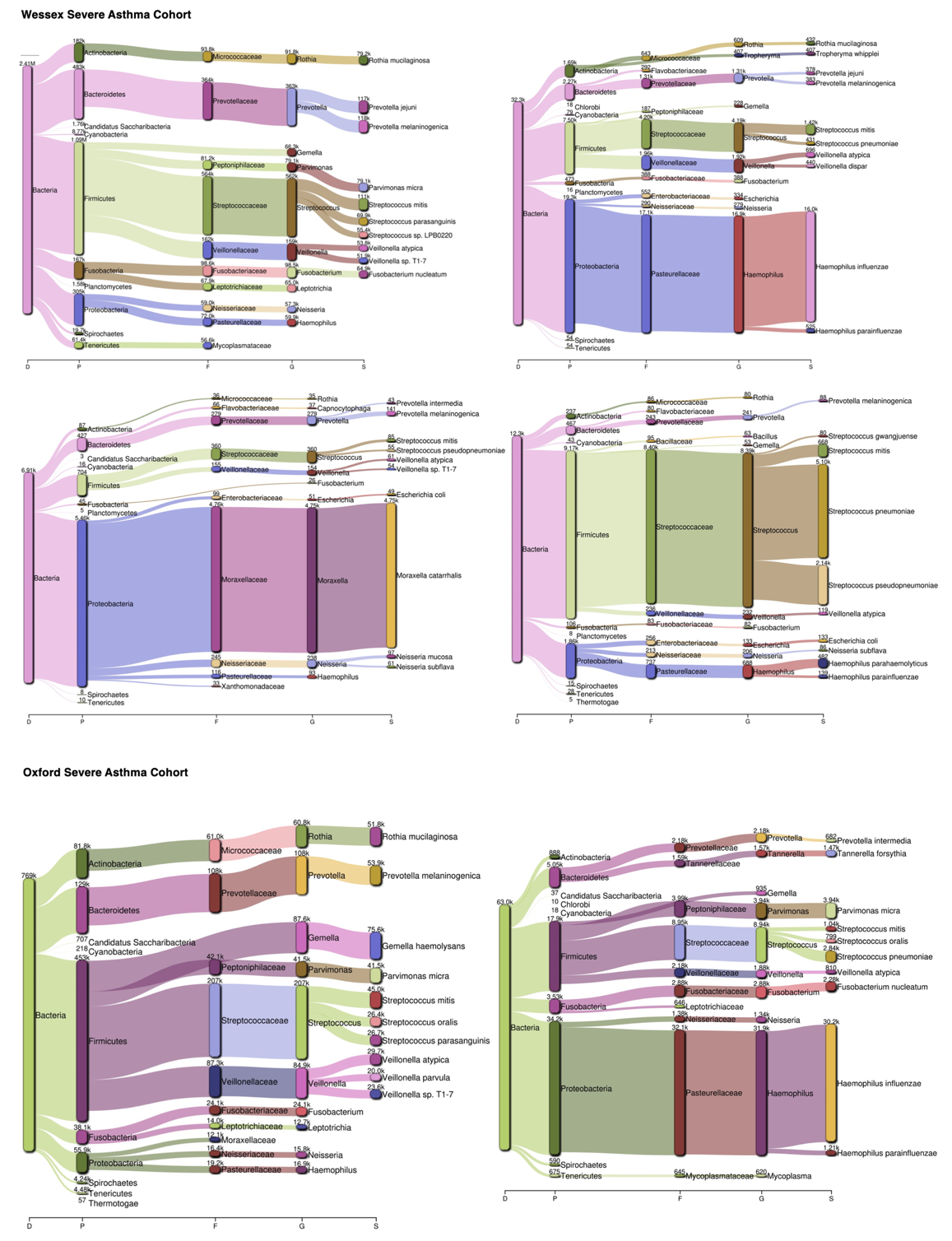


(D)

(F)

(E)

(C)

(A)

(B)

(G)

(H)


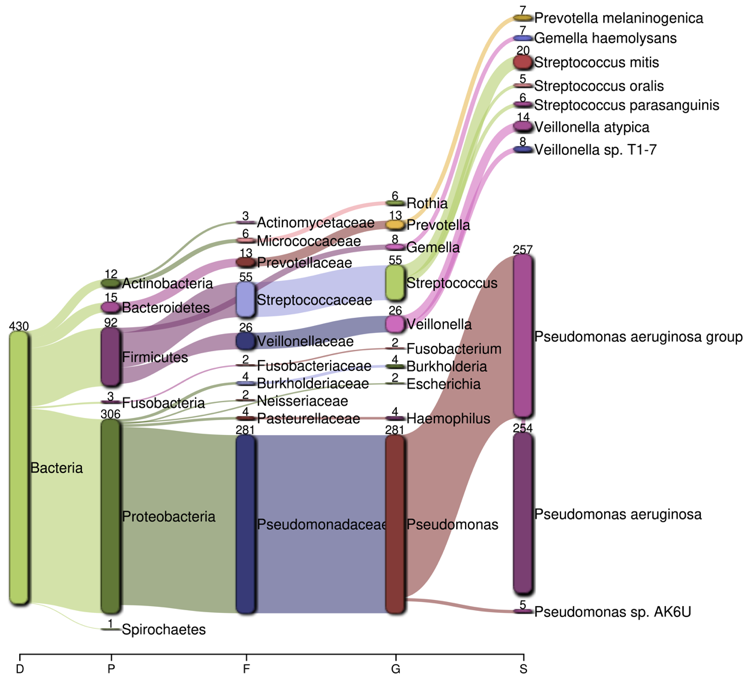


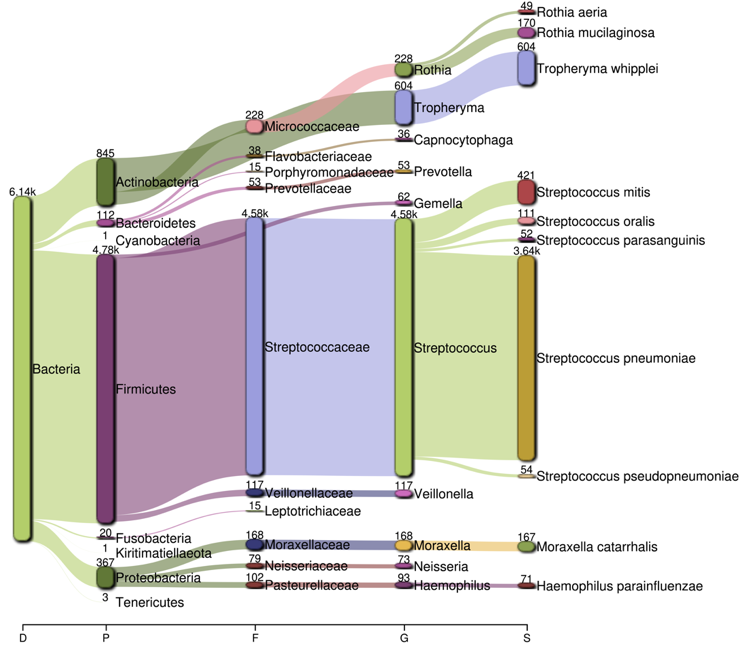


## **Table E3 Top species in health**

Top species in Healthy controls (n=5) vs Mild and severe asthmatics (n=61); Wald test used to identify uniquely abundant species using DESeq2, log fold change > 1 and adjusted P value < 0.05 following adjustment for multiple comparisons using Benjamini–Hochberg procedure (FDR 0.05). LFC, log fold change; SE, standard error.

| **Species** | **Base Mean** | **LFC** | **LFC (SE)** | **Wald statistic** | **P value** | **Adjusted P value** |
| --- | --- | --- | --- | --- | --- | --- |
| *Streptococcus viridans* | 163.47 | 6.28 | 0.83 | 7.61 | 2.67E-14 | 6.77E-12 |
| *Streptococcus sp. A12* | 184.19 | 6.09 | 0.83 | 7.30 | 2.80E-13 | 3.54E-11 |
| *Streptococcus australis* | 208.41 | 5.04 | 0.75 | 6.74 | 1.59E-11 | 1.34E-09 |
| *Schaalia odontolytica* | 997.00 | 8.51 | 1.28 | 6.64 | 3.11E-11 | 1.97E-09 |
| *Schaalia meyeri* | 44.38 | 7.67 | 1.19 | 6.43 | 1.26E-10 | 6.38E-09 |
| *Lancefieldella parvula* | 886.86 | 6.79 | 1.07 | 6.37 | 1.86E-10 | 7.83E-09 |
| *Actinomyces pacaensis* | 146.66 | 6.29 | 1.02 | 6.14 | 8.00E-10 | 2.25E-08 |
| *Streptococcus intermedius* | 52.51 | 4.41 | 0.79 | 5.61 | 2.08E-08 | 4.79E-07 |
| *Mogibacterium diversum* | 535.90 | 5.97 | 1.14 | 5.24 | 1.61E-07 | 3.39E-06 |
| *Streptococcus gordonii* | 168.13 | 4.04 | 0.79 | 5.12 | 3.05E-07 | 5.52E-06 |
| *Streptococcus anginosus group* | 116.19 | 3.47 | 0.79 | 4.42 | 9.99E-06 | 1.10E-04 |
| *Gemella sanguinis* | 216.90 | 3.16 | 0.89 | 3.56 | 3.66E-04 | 2.11E-03 |
| *Streptococcus parasanguinis* | 580.65 | 2.91 | 0.88 | 3.32 | 9.03E-04 | 4.20E-03 |
| *Prevotella oris* | 200.84 | 2.48 | 0.75 | 3.31 | 9.21E-04 | 4.20E-03 |
| *Leptotrichia sp. oral taxon 212* | 104.88 | 3.03 | 0.92 | 3.31 | 9.18E-04 | 4.20E-03 |
| *Streptococcus sp. LPB0220* | 450.84 | 2.72 | 0.85 | 3.19 | 1.44E-03 | 5.93E-03 |
| *Streptococcus sp. oral taxon 431* | 209.08 | 2.25 | 0.75 | 3.00 | 2.68E-03 | 9.95E-03 |
| *Streptococcus sp. oral taxon 061* | 198.59 | 2.29 | 0.77 | 2.97 | 3.01E-03 | 1.09E-02 |
| *Streptococcus anginosus* | 50.74 | 2.49 | 0.88 | 2.83 | 4.72E-03 | 1.55E-02 |
| *Gemella morbillorum* | 64.14 | 2.38 | 0.88 | 2.69 | 7.22E-03 | 2.17E-02 |
| *Leptotrichia wadei* | 182.75 | 2.30 | 0.97 | 2.37 | 1.79E-02 | 4.38E-02 |

## **Table E4 Top species in mild asthma**

Top species in Mild asthma (n=10) vs. Healthy controls and Severe asthma (n=56); Wald test used to identify uniquely abundant species using DESeq2, log fold change > 1 and adjusted P value < 0.05 following adjustment for multiple comparisons using Benjamini–Hochberg procedure (FDR 0.05). LFC, log fold change; SE, standard error.

| **Species** | **Base Mean** | **LFC** | **LFC (SE)** | **Wald statistic** | **P value** | **Adjusted P value** |
| --- | --- | --- | --- | --- | --- | --- |
| *Filifactor alocis* | 46.16 | 2.64 | 0.78 | 3.37 | 7.38E-04 | 9.83E-03 |
| *Mogibacterium diversum* | 535.90 | 2.53 | 0.79 | 3.21 | 1.32E-03 | 1.52E-02 |
| *Streptococcus sp. A12* | 184.19 | 1.69 | 0.59 | 2.84 | 4.47E-03 | 4.04E-02 |
| *Streptococcus sp. oral taxon 061* | 198.59 | 1.45 | 0.55 | 2.64 | 8.41E-03 | 6.88E-02 |
| *Streptococcus salivarius* | 268.76 | 1.82 | 0.70 | 2.62 | 8.87E-03 | 7.01E-02 |
| *Streptococcus viridans* | 163.47 | 1.53 | 0.59 | 2.58 | 9.93E-03 | 7.62E-02 |
| *Streptococcus intermedius* | 52.51 | 1.15 | 0.58 | 1.98 | 4.72E-02 | 2.91E-01 |
| *Streptococcus australis* | 208.41 | 1.07 | 0.55 | 1.95 | 5.14E-02 | 3.09E-01 |

## **Table E5 Top species in severe asthma**

Most abundant species and species most depleted in severe asthma (n=51) vs Healthy controls and Mild asthma (n=15); Wald test used to identify species uniquely abundant or depleted in SA using DESeq2, log fold change [LFC] > 1 or <1 respectively, adjusted P value < 0.05 following adjustment for multiple comparisons using Benjamini–Hochberg procedure (FDR 0.05). LFC, log fold change; SE, standard error.

| Most abundant species | | | | | | |
| --- | --- | --- | --- | --- | --- | --- |
| **Species** | **Base Mean** | **LFC** | **LFC (SE)** | **Wald statistic** | **P value** | **Adjusted P value** |
| *Rothia mucilaginosa* | 162.27 | 3.75 | 0.46 | 8.16 | 3.44E-16 | 8.38E-14 |
| *Rothia dentocariosa* | 52.98 | 3.10 | 0.60 | 5.11 | 3.22E-07 | 1.12E-05 |
| *Haemophilus influenzae* | 212.03 | 3.09 | 0.68 | 4.58 | 4.73E-06 | 8.21E-05 |
| *Moraxella catarrhalis* | 40.25 | 4.30 | 1.18 | 3.66 | 2.57E-04 | 2.01E-03 |
| *Streptococcus pseudopneumoniae* | 86.78 | 1.50 | 0.55 | 2.72 | 6.46E-03 | 2.45E-02 |
| Most depleted species | | | | | | |
| **Species** | **Base Mean** | **LFC** | **LFC (SE)** | **Wald statistic** | **P value** | **Adjusted P value** |
| *Streptococcus anginosus group* | 116.19 | -2.29 | 0.53 | -4.30 | 1.67E-05 | 2.54E-04 |
| *Streptococcus sp. oral taxon 061* | 198.59 | -1.97 | 0.47 | -4.18 | 2.93E-05 | 3.75E-04 |
| *Gemella sanguinis* | 216.90 | -2.24 | 0.57 | -3.90 | 9.69E-05 | 1.18E-03 |
| *Streptococcus parasanguinis* | 580.65 | -2.13 | 0.56 | -3.82 | 1.35E-04 | 1.42E-03 |
| *Mogibacterium diversum* | 100.49 | -2.32 | 0.62 | -3.74 | 1.86E-04 | 1.70E-03 |
| *Filifactor alocis* | 46.16 | -2.48 | 0.66 | -3.73 | 1.88E-04 | 1.70E-03 |
| *Streptococcus sp. oral taxon 431* | 209.08 | -1.67 | 0.47 | -3.52 | 4.38E-04 | 3.13E-03 |
| *Streptococcus sp. LPB0220* | 450.84 | -1.89 | 0.55 | -3.46 | 5.44E-04 | 3.70E-03 |
| *Streptococcus salivarius* | 268.76 | -1.94 | 0.59 | -3.28 | 1.04E-03 | 5.51E-03 |
| *Lachnoanaerobaculum umeaense* | 70.48 | -1.64 | 0.59 | -2.81 | 4.99E-03 | 2.15E-02 |
| *Lancefieldella parvula* | 103.29 | -1.61 | 0.58 | -2.80 | 5.14E-03 | 2.15E-02 |
| *Leptotrichia wadei* | 182.75 | -1.51 | 0.62 | -2.43 | 1.51E-02 | 5.01E-02 |
| *Streptococcus anginosus* | 50.74 | -1.40 | 0.58 | -2.41 | 1.61E-02 | 5.21E-02 |

## **Table E6 Single pathogen dominance in severe asthma**

Dominance of a single pathogen in severe asthma

| **Cohort** | **PID†** | **Dominant Pathogen** | **%Pathogen/ bacterial reads‡** | **Length of reference genome (bases)** | **Coverage breadth of reference genome (%)§** | **Average coverage depth¶** | **Pathogen specific PCR (x10^6^ copies/ml)ll** | **Sputum inflammatory phenotype** | **Clinical sputum culture result** |  |
| --- | --- | --- | --- | --- | --- | --- | --- | --- | --- | --- |
|  |  |  |  |  |  |  |  |  |  |  |
| Oxford | OAS1076 | *Haemophilus influenzae* | 77 | 1.83E+06 | 92.9 | 11.0 | **179.9** | Neutrophilic | *H. influenzae* |  |
| Oxford | OAS1021 | *Haemophilus influenzae* | 93 | 1.83E+06 | 74.1 | 2.1 | **175.4** | Neutrophilic | URT flora |  |
| Oxford | OAS1102 | *Haemophilus influenzae* | 92 | 1.83E+06 | 89.9 | 8.0 | **135.6** | Neutrophilic | *H. influenzae* |  |
| Oxford | OAS1145 | *Haemophilus influenzae* | 22 | 1.83E+06 | 88.6 | 4.5 | **83.9** | ND | ND |  |
| Oxford | OAS1041 | *Moraxella catarrhalis* | 59 | 1.86E+06 | 19.1 | 3.8 | **4.3** | Neutrophilic | URT flora |  |
| Oxford | OAS1047 | *Streptococcus pneumoniae* | 57 | 2.04E+06 | 83.0 | 2.6 | **28.3** | Neutrophilic | *S. pneumoniae* |  |
| Oxford | OAS1065 | *Streptococcus pneumoniae* | 63 | 2.04E+06 | 56.5 | 1.7 | **15.9** | ND | *S. pneumoniae* |  |
| Oxford | OAS1017 | *Pseudomonas aeruginosa* | 73 | 6.26E+06 | 5.1 | 1.0 | **4.6** | ND | URT flora |  |
| Wessex | MRCSAC0142 | *Haemophilus influenzae* | 78 | 1.83E+06 | 92.6 | 14.5 | **6.2** | Neutrophilic | ND |  |
| Wessex | MRCSAC0115 | *Haemophilus influenzae* | 54 | 1.83E+06 | 52.8 | 1.5 | **5.7** | Neutrophilic | ND |  |
| Wessex | MRCSAC0130 | *Haemophilus influenzae* | 17 | 1.83E+06 | 65.5 | 2.0 | **4.8** | Mixed | ND |  |
| Wessex | MRCSAC0098 | *Haemophilus influenzae* | 93 | 1.83E+06 | 88.8 | 3.5 | **2.1** | Neutrophilic | ND |  |
| Wessex | MRCSAC0151 | *Haemophilus influenzae* | 25 | 1.83E+06 | 47.3 | 1.5 | 0.8 | Eosinophilic | ND |  |
| Wessex | MRCSAC0150 | *Haemophilus influenzae* | 72 | 1.83E+06 | 20.0 | 1.2 | 0.5 | Eosinophilic | ND |  |
| Wessex | MRCSAC0101 | *Moraxella catarrhalis* | 79 | 1.86E+06 | 95.2 | 4.3 | **33.8** | Eosinophilic | ND |  |
| Wessex | MRCSAC0132 | *Moraxella catarrhalis* | 92 | 1.86E+06 | 92.6 | 3.7 | **18.8** | Neutrophilic | ND |  |
| Wessex | MRCSACC001 | *Moraxella catarrhalis* | 28 | 1.86E+06 | 23.5 | 1.2 | **18.6** | Neutrophilic | ND |  |
| Wessex | MRCSAC0135 | *Streptococcus pneumoniae* | 93 | 2.04E+06 | 92.8 | 4.3 | **18.7** | Paucigranulocytic | ND |  |
| Wessex | MRCSAC0104 | *Streptococcus pneumoniae* | 79 | 2.04E+06 | 79.0 | 2.4 | 0.7 | Neutrophilic | ND |  |

†Patients with severe asthma from Wessex and Oxford cohorts demonstrating dominance of single pathogenic organism using Nanopore. ‡Proportion of total bacterial reads occupied by indicated pathogenic organism (represented as bar chart). §Coverage breadth (percentage of reference genome covered, represented as bar chart). ¶Average coverage depth (at positions with ≥1 read). llClinically significant (≥1x10^6^ copies/ml) PCR result shown in bold. Inflammatory phenotypes: eosinophilic >3% sputum eosinophils, neutrophilic >61% sputum neutrophils and <3% eosinophils, mixed granulocytic >61% sputum neutrophils and >3% eosinophils, paucigranulocytic <61% sputum neutrophils and <3% eosinophils. ND, no data (not performed)

†Patients with severe asthma from Wessex and Oxford cohorts demonstrating dominance of single pathogenic organism using Nanopore. ‡Proportion of total bacterial reads occupied by indicated pathogenic organism (represented as bar chart). §Coverage breadth (percentage of reference genome covered, represented as bar chart). ¶Average coverage depth (at positions with ≥1 read). llClinically significant (≥1x10^6^ copies/ml) PCR result shown in bold. Inflammatory phenotypes: eosinophilic >3% sputum eosinophils, neutrophilic >61% sputum neutrophils and <3% eosinophils, mixed granulocytic >61% sputum neutrophils and >3% eosinophils, paucigranulocytic <61% sputum neutrophils and <3% eosinophils. ND, no data (not performed)

## **Table E7 Species by inflammatory phenotype**

Most differentially abundant bacterial species in severe asthma by inflammatory phenotype; Neutrophilic, Eosinophilic, and Paucigranulocytic. Wald test used to identify species uniquely abundant or depleted relative to other inflammatory phenotypes (using DESeq2, adjusted P < 0.05 following adjustment for multiple comparisons using Benjamini–Hochberg procedure [FDR 0.05]).

| Neutrophilic | | | | | | |
| --- | --- | --- | --- | --- | --- | --- |
| **Species** | **Base Mean** | **LFC** | **LFC (SE)** | **Wald statistic** | **P value** | **Adjusted P value** |
| *Haemophilus influenzae* | 3568.22 | 6.70 | 0.70 | 9.58 | 1.01E-21 | 7.05E-20 |
| *Moraxella catarrhalis* | 601.35 | 8.18 | 0.88 | 9.30 | 1.40E-20 | 4.88E-19 |
| *Tropheryma whipplei* | 61.31 | 4.56 | 0.70 | 6.55 | 5.76E-11 | 1.34E-09 |
| *Streptococcus pneumoniae* | 779.90 | 3.65 | 0.63 | 5.76 | 8.30E-09 | 1.45E-07 |
| *Streptococcus salivarius* | 230.80 | -2.26 | 0.49 | -4.63 | 3.59E-06 | 5.03E-05 |
| *Streptococcus intermedius* | 29.90 | -1.31 | 0.41 | -3.17 | 1.52E-03 | 1.77E-02 |
| *Streptococcus sp. LPB0220* | 315.57 | -1.39 | 0.46 | -3.06 | 2.19E-03 | 1.91E-02 |
| *Streptococcus sp. oral taxon 431* | 110.11 | -0.96 | 0.31 | -3.06 | 2.18E-03 | 1.91E-02 |
| *Streptococcus parasanguinis* | 366.46 | -1.23 | 0.43 | -2.84 | 4.56E-03 | 2.90E-02 |
| *Streptococcus australis* | 64.02 | -0.96 | 0.34 | -2.86 | 4.18E-03 | 2.90E-02 |
| *Haemophilus haemolyticus* | 44.71 | 1.25 | 0.44 | 2.84 | 4.46E-03 | 2.90E-02 |
| *Streptococcus oralis* | 187.95 | -0.94 | 0.35 | -2.71 | 6.80E-03 | 3.40E-02 |
| *Streptococcus anginosus group* | 60.38 | -0.97 | 0.36 | -2.73 | 6.32E-03 | 3.40E-02 |
| *Prevotella enoeca* | 20.17 | 0.76 | 0.28 | 2.72 | 6.55E-03 | 3.40E-02 |
| Eosinophilic | | | | | | |
| **Species** | **Base Mean** | **LFC** | **LFC (SE)** | **Wald statistic** | **P value** | **Adjusted P value** |
| *Streptococcus pneumoniae* | 779.90 | -3.74 | 0.63 | -5.89 | 3.76E-09 | 2.63E-07 |
| *Moraxella catarrhalis* | 601.35 | 4.07 | 0.97 | 4.18 | 2.90E-05 | 1.02E-03 |
| *Streptococcus intermedius* | 29.90 | 1.33 | 0.40 | 3.36 | 7.76E-04 | 1.48E-02 |
| *Actinomyces pacaensis* | 36.49 | -1.66 | 0.50 | -3.34 | 8.49E-04 | 1.48E-02 |
| *Haemophilus influenzae* | 3568.22 | -2.81 | 0.86 | -3.27 | 1.06E-03 | 1.48E-02 |
| *Veillonella parvula* | 189.94 | 1.12 | 0.37 | 3.02 | 2.49E-03 | 2.90E-02 |
| Paucigranulocytic | | | | | | |
| **Species** | **Base Mean** | **LFC** | **LFC (SE)** | **Wald statistic** | **P value** | **Adjusted P value** |
| *Moraxella catarrhalis* | 601.35 | -8.57 | 0.80 | -10.76 | 5.55E-27 | 3.61E-25 |
| *Haemophilus influenzae* | 3568.22 | -5.96 | 0.74 | -8.01 | 1.12E-15 | 3.65E-14 |
| *Tropheryma whipplei* | 61.31 | -5.04 | 0.72 | -7.05 | 1.85E-12 | 4.00E-11 |
| *Mogibacterium diversum* | 123.71 | 2.00 | 0.47 | 4.27 | 1.95E-05 | 3.17E-04 |
| *Streptococcus pneumoniae* | 779.90 | 2.42 | 0.65 | 3.75 | 1.76E-04 | 1.91E-03 |
| *Treponema sp. OMZ 804* | 17.40 | 1.67 | 0.44 | 3.77 | 1.65E-04 | 1.91E-03 |
| *Streptococcus australis* | 64.02 | 1.06 | 0.30 | 3.47 | 5.14E-04 | 4.78E-03 |
| *Gemella morbillorum* | 42.12 | 1.11 | 0.34 | 3.26 | 1.12E-03 | 9.08E-03 |
| *Streptococcus gordonii* | 85.98 | 1.17 | 0.37 | 3.16 | 1.60E-03 | 1.15E-02 |
| *Schaalia odontolytica* | 156.13 | 1.76 | 0.56 | 3.11 | 1.84E-03 | 1.20E-02 |
| *Actinomyces pacaensis* | 36.49 | 1.39 | 0.45 | 3.06 | 2.23E-03 | 1.31E-02 |
| *Lachnoanaerobaculum umeaense* | 42.21 | 0.98 | 0.35 | 2.80 | 5.18E-03 | 2.59E-02 |
| *Prevotella jejuni* | 285.63 | -1.28 | 0.45 | -2.81 | 4.93E-03 | 2.59E-02 |
| *Streptococcus sp. oral taxon 431* | 110.11 | 0.82 | 0.30 | 2.70 | 6.84E-03 | 3.17E-02 |
| *Filifactor alocis* | 33.44 | 1.14 | 0.42 | 2.68 | 7.31E-03 | 3.17E-02 |
| *Veillonella sp. T1-7* | 299.93 | -0.92 | 0.35 | -2.61 | 9.15E-03 | 3.72E-02 |
| *Streptococcus anginosus* | 29.86 | 0.86 | 0.34 | 2.56 | 1.05E-02 | 4.01E-02 |

## **Figure E5 Sankey visualisations in Wessex Cohort**

Sankey visualisation of taxonomic profiles in Wessex Cohort generated using Pavian.; (a) Healthy controls, (b) Mild asthma.


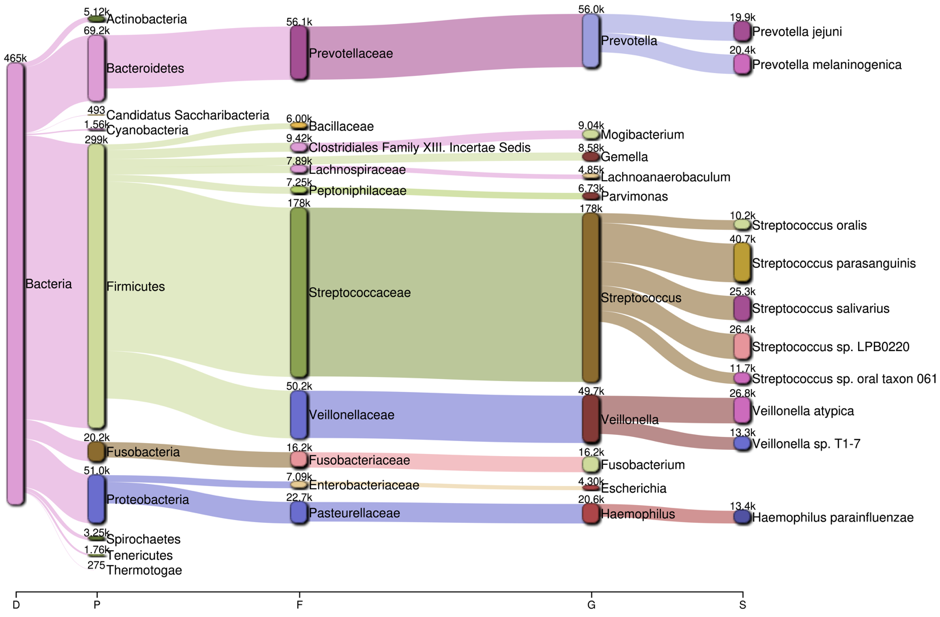


(a)

(b)


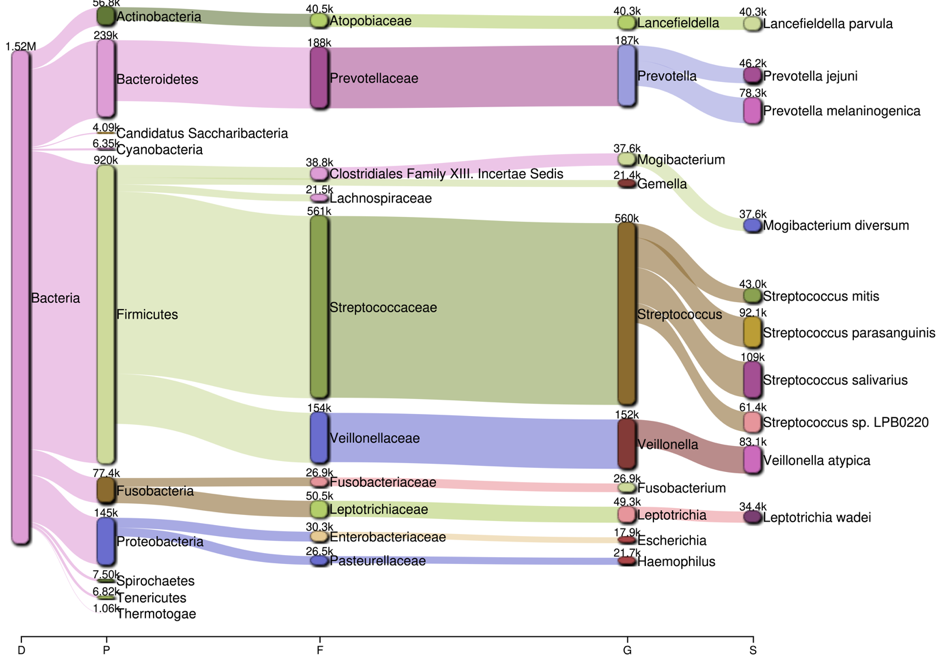


## **Figure E6 Proteobacteria:firmicute ratios**

Ratio of Proteobacteria:Firmicute reads, grouped by: (a) Disease severity and cohort, (b) Absence or presence of airways infection, (c) Inflammatory phenotype (neutrophilic, n=22; eosinophilic, n=19; mixed, n=6; paucigranulocytic, n=24). Median/IQR shown, unpaired t-test, ns >0.05, ***P≤0.001). Data points in maroon indicate presence of airways infection.

(b)

(a)


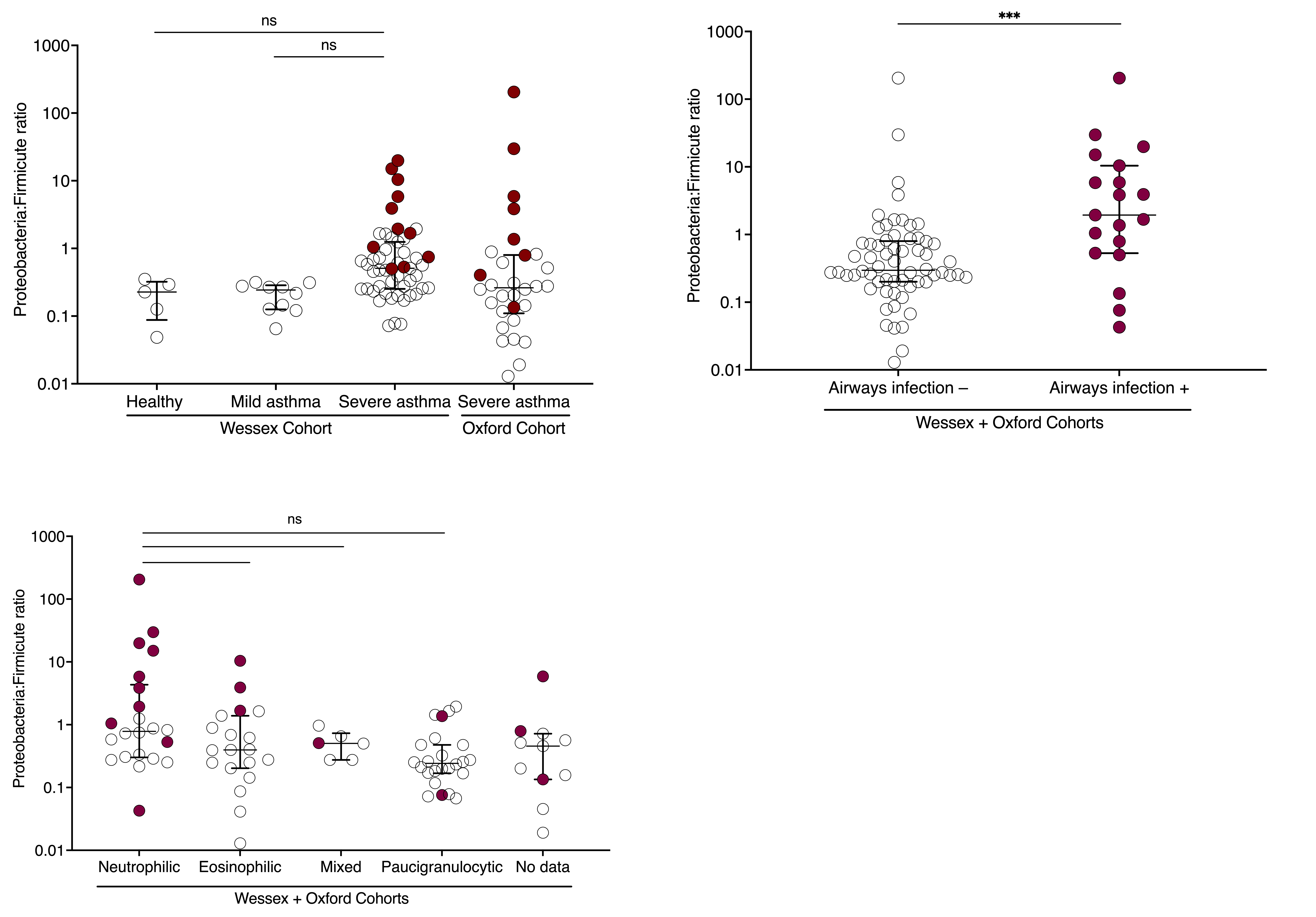


(c)

## **Table E8 Sputum cytokines by presence of infection**

Comparison of sputum cytokines in the presence or absence of airways infection in severe asthma; Median/IQR shown −/+Airways infection alongside adjusted P value (unpaired t-test, adjusted for multiple comparisons with Benjamini–Hochberg procedure [FDR 0.05])

|  | **Airways infection absent** | | **Airways infection present** | |  |
| --- | --- | --- | --- | --- | --- |
| **Cytokine (pg/ml)** | Median | IQR | Median | IQR | **Adjusted P value** |
| Elastase | 2530 | 2892 | 8414 | 8515 | 0.002 |
| TNF | 0 | 0 | 25 | 104 | 0.002 |
| IL-10 | 0 | 0 | 3 | 6 | 0.010 |
| IL-1β | 28 | 104 | 435 | 725 | 0.010 |
| MIP-1β | 29 | 239 | 334 | 521 | 0.010 |
| MMP8 | 122066 | 260721 | 436658 | 143419 | 0.010 |
| ECP | 118 | 615 | 2333 | 5154 | 0.010 |
| YKL40 | 40 | 74 | 204 | 225 | 0.010 |
| MPO | 1349 | 4046 | 7288 | 5774 | 0.014 |
| IL-8 | 1611 | 3650 | 9760 | 6512 | 0.018 |
| MIP-1α | 8 | 183 | 342 | 279 | 0.019 |
| IL-6SR | 181 | 209 | 396 | 218 | 0.019 |
| MMP9 | 78429 | 74121 | 215031 | 174057 | 0.020 |
| TIMP1 | 324 | 432 | 464 | 374 | 0.044 |
| IL-1RA | 10689 | 7656 | 0 | 8500 | 0.045 |
| IFNɣ | 0 | 0 | 0 | 2 | 0.050 |
| IL-17 | 0 | 1 | 0 | 1 | 0.053 |
| IL-6 | 29 | 76 | 128 | 156 | 0.079 |
| ENA78 | 231 | 326 | 400 | 424 | 0.079 |
| GCSF | 209 | 293 | 420 | 549 | 0.105 |
| MMP3 | 322 | 1032 | 642 | 969 | 0.190 |
| VEGF | 588 | 535 | 970 | 515 | 0.192 |
| MCP1 | 150 | 222 | 458 | 287 | 0.208 |
| IL-5 | 0 | 1 | 2 | 16 | 0.216 |
| GMCSF | 0 | 0 | 0 | 1 | 0.221 |
| MMP7 | 52794 | 111539 | 98919 | 112724 | 0.282 |
| α2-macroglobulin | 116 | 275 | 275 | 537 | 0.300 |
| IL-1α | 8 | 20 | 27 | 82 | 0.324 |
| Tryptase | 1 | 4 | 2 | 6 | 0.336 |
| MMP1 | 172 | 125 | 181 | 253 | 0.439 |
| Eotaxin | 13 | 66 | 48 | 37 | 0.489 |
| IL-13 | 0 | 0 | 0 | 0 | 0.502 |
| IL-4 | 0 | 0 | 0 | 0 | 0.570 |
| MMP2 | 2756 | 4092 | 2732 | 3886 | 0.570 |
| MMP12 | 183 | 144 | 144 | 147 | 0.570 |
| OPN | 23231 | 17016 | 18358 | 23398 | 0.656 |
| GROα | 288 | 1092 | 770 | 2457 | 0.695 |
| MMP13 | 888 | 607 | 426 | 727 | 0.785 |
| FGF | 0 | 0 | 0 | 0 | 0.799 |

## **Table E9 Bayesian Network Analysis variables**

BNA variables; 60 variables selected including key clinical variables, 15 cytokines (showing statistically significant differences in absence and presence of airways infection) and 25 bacterial species (most differentially abundant in severe asthma). These variables were discretised into 2 (binary variables) or 3 to 5 (continuous variables) bins. Variables retained in the model are shown in bold.

| Demographic | | | |
| --- | --- | --- | --- |
| Sex | Age (years) | Asthma duration (years) | 2  BMI (kg/m ) |
| Clinical parameters | | | |
| BTS step Nasal Polyps  ACQ7 | ICS dose (BDP equivalent, mcg/d) Rhinosinusitis  **Infection Status** | Macrolide use Cough | Atopy  Smoking History |
| Pulmonary function | | | |
| **FeNO (ppb)** | **FEV1(%)** | **FEV1/FVC** |  |
| Peripheral blood | | | |
| 9  Blood eosinophils (x10 /L) | | | |
| Sputum inflammation | | | |
| Sputum neutrophils (%) | Sputum eosinophils (%) | **Sputum IL-10** | Sputum IL-1RA |
| **Sputum IL-1β** | **Sputum TNF** | **Sputum Elastase** | **Sputum MIP1β** |
| Sputum MMP8 | **Sputum ECP** | **Sputum YKL40** | **Sputum MPO** |
| **Sputum IL-8** | **Sputum MIP1α** | **Sputum IL-6SR** | Sputum MMP9 |
| **Sputum TIMP1** |  |  |  |
| Sputum microbiology | | | |
| *Streptococcus parasanguinis* | ***Streptococcus viridans*** | *Rothia mucilaginosa* | ***Rothia dentocariosa*** |
| *Prevotella oris* | ***Haemophilus influenzae*** | ***Moraxella catarrhalis*** | ***Neisseria subflava*** |
| ***Neisseria mucosa*** | ***Streptococcus sp. oral taxon 061*** | *Streptococcus sp. LPB0220* | ***Streptococcus sp. oral taxon 431*** |
| ***Streptococcus sp. A12*** | ***Streptococcus australis*** | ***Streptococcus gordonii*** | ***Streptococcus anginosus group*** |
| ***Streptococcus intermedius*** | *Gemella sanguinis* | *Mogibacterium diversum* | ***Filifactor alocis*** |
| ***Actinomyces pacaensis*** | ***Schaalia odontolytica*** | ***Schaalia meyeri*** | *Lancefieldella parvula* |
| ***Leptotrichia sp. oral taxon 212*** |  |  |  |

## **Figure E7 Correlations between inflammatory mediators**


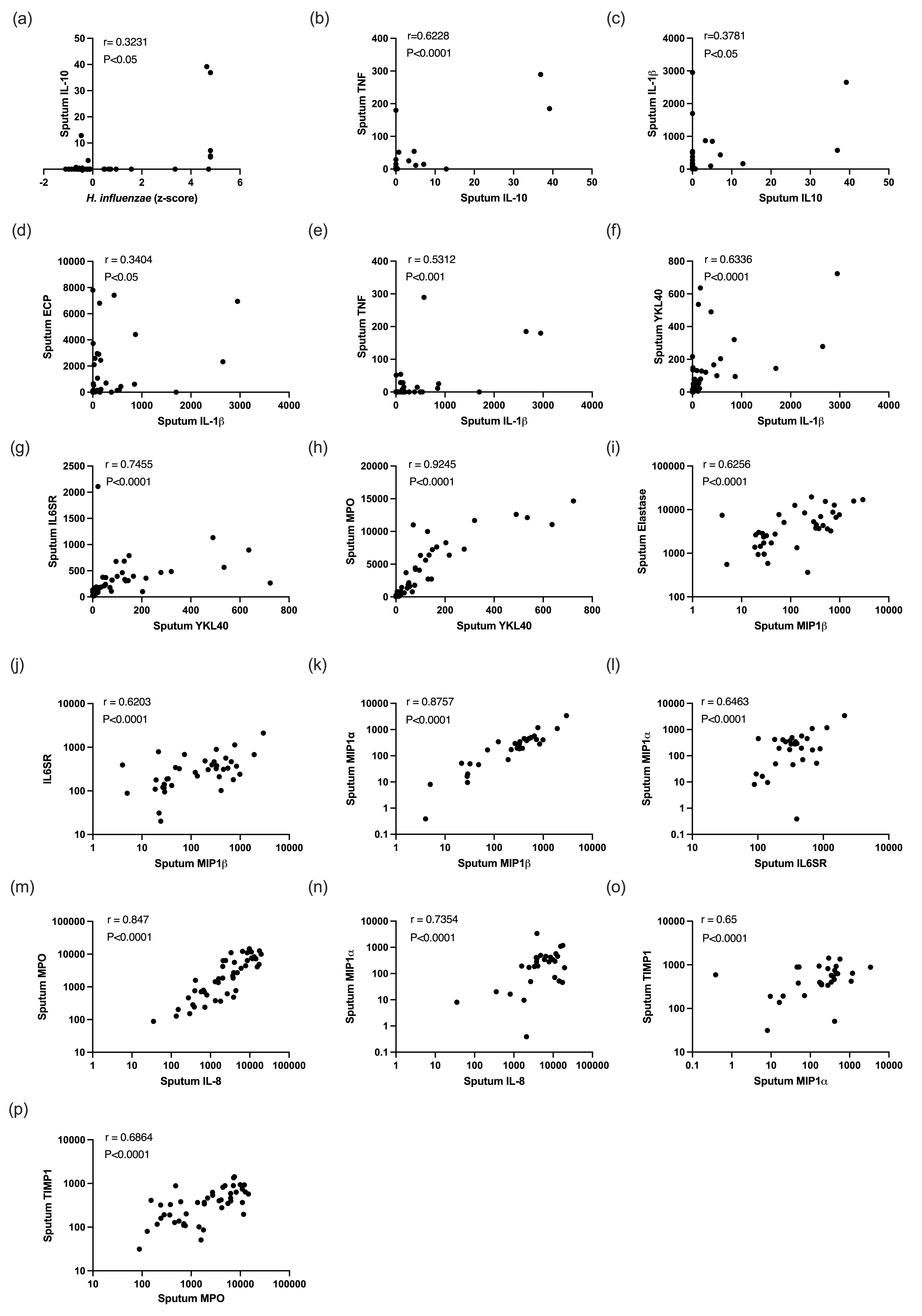
Microbial and inflammatory mediators shown to be associated through BNA. Spearman correlations shown for pairwise interactions identified in BNA. Correlations were tested with Spearman R statistic

##

## **Figure E8 Relationship of FeNO to *H. influenzae* abundance**

Scatter plot of relationship between *H influenzae* qPCR quantification (copies/ml) and fractional exhaled nitric oxide (FeNO) in parts per billion (ppb).


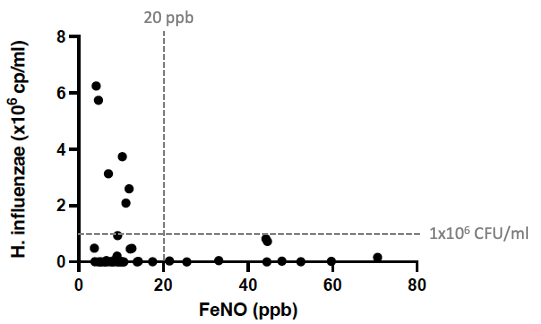


## **Figure E9 Pairwise associations between bacterial species**

Bacteria shown to be associated through BNA. Pearson correlations shown for pairwise interactions identified in BNA. Correlations were tested with Pearson R statistic


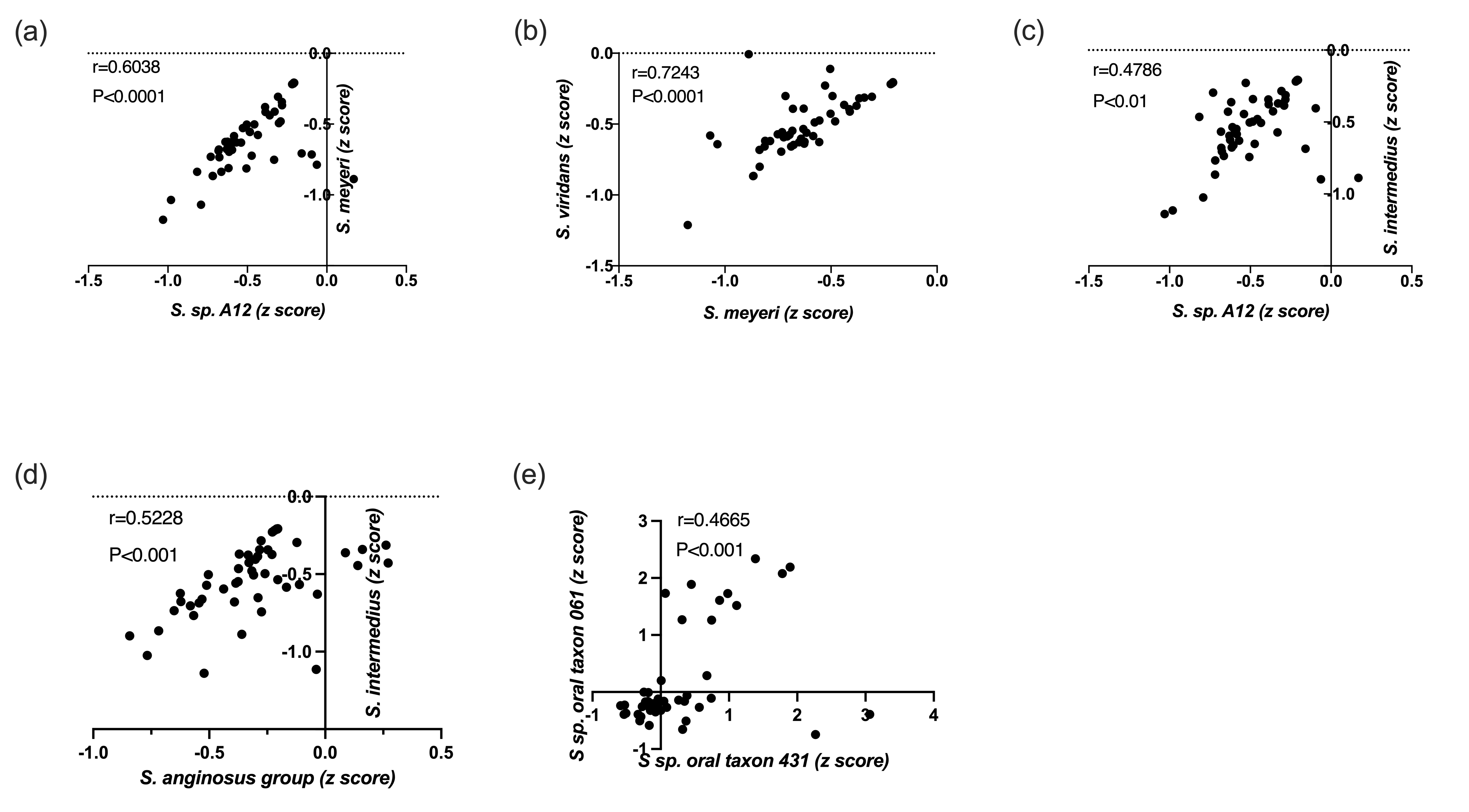


## **Figure E10 Upper and lower airway microbiome and cytokines**

Heatmap of relative abundances of bacterial species on metagenomic sequencing of paired nasal lavage (NL) and sputum (SP) samples from severe asthmatics (n=17). Samples have been arranged to show paired upper and lower airway samples in adjacent columns for comparison. Z-score denoted by shade. Independent clustering by 23 most variable species (rows) using Euclidian distance.

**Upper and lower airway cytokine milieu is distinct in severe asthma**

The cytokine milieu in the upper and lower airway was compared using paired O-link proteomic data from nasal lavage and sputum samples. The most significantly correlated (P<<0.001) proteins are displayed as a correlation matrix in Figure 5(b). There is a separation between nasal lavage and sputum compartments (that is, proteins most strongly correlate with others from the same sample type). The exceptions to this are two clusters – the first showing strong positive correlation between sputum cytokines IL-20, FGF5 and IL-2 with nasal lavage proteins neurotrophin 3, neurturin, FGF23, IL-5, FGF5, IL-10Rα, nerve growth factor (NGF) and IL-20. The second cluster shows a negative correlation between the sputum cytokines IL-20, FGF5 and IL-2, and nasal lavage proteins urokinase, herpesvirus entry mediator ligand (HVEML), adenosine deaminase (ADA), oncostatin M, hepatocyte growth factor (HGF), S100A12, CCL44, CCL3, IL-18R1, CD318, tumour necrosis factor-like weak inducer of apoptosis (TWEAK), MMP10, VEGFA and CD40.

## **Figure E11 PCA of metagenomic profiles**

Principal component analysis (PCA) based on metagenomic profiles with convex hulls plotted for paired nasal (pink) and sputum (yellow) samples derived from patients with severe asthma from the Wessex Severe Asthma Cohort (PERMANOVA = 0.001). First two principal components are shown.


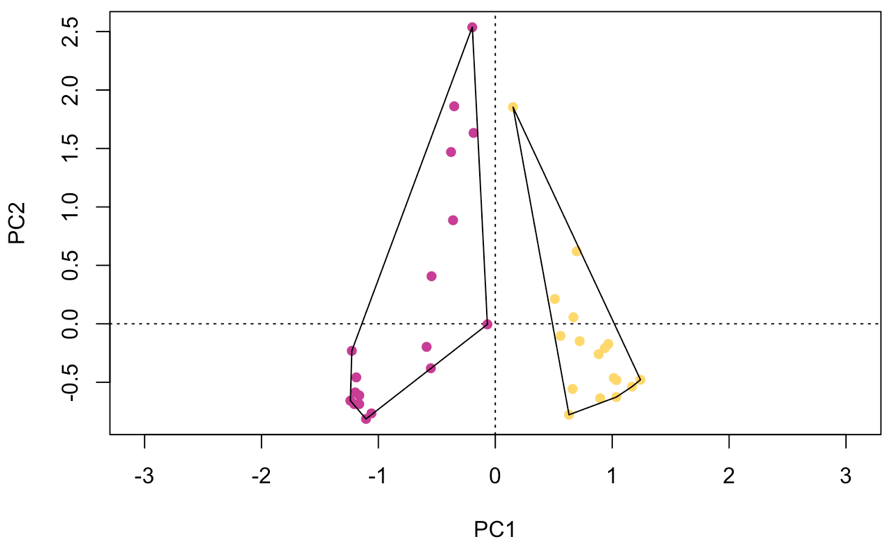

Supplement: Online supplement [file EMS206154-supplement-Online_supplement.docx]
